# Effects of post-exercise stretching versus no stretching on lower limb muscle recovery and performance: a meta-analysis

**DOI:** 10.3389/fphys.2025.1674871

**Published:** 2025-10-01

**Authors:** Pei Zhang, Jiangzhou Chen, Taofeng Xing

**Affiliations:** ^1^ Program in Global Exercise Science, Arts and Sports College, Inha University, Incheon, Republic of Korea; ^2^ Department of Health and Exercise Science, College of Graduate School, Inha University, Incheon, Republic of Korea; ^3^ Department of Sports and Leisure, College of Humanity, Daegu University, Gyeongsan, Republic of Korea

**Keywords:** post-exercise stretching, muscle recovery, athletic performance, athletic adaptation, meta-analysis

## Abstract

**Background:**

Post-exercise stretching is widely employed in athletic and rehabilitation settings to promote recovery and performance. However, its physiological benefits remain controversial due to inconsistent findings across randomized controlled trials.

**Objective:**

To evaluate the effects of post-exercise stretching compared to no stretching on lower limb muscle recovery and performance indicators, including muscle soreness, strength, flexibility, performance, and pain threshold.

**Methods:**

A systematic search was conducted in eight databases up to 20 July 2025. Randomized controlled trials, controlled clinical trials, and crossover trials comparing post-exercise stretching (static, dynamic, or PNF) with no stretching were included. Data were synthesized using random-effects models, and effect sizes were expressed as standardized mean differences (SMDs). Risk of bias was assessed using the Cochrane RoB 2.0 tool.

**Results:**

Fifteen studies (n = 465 participants) were included. Post-exercise stretching showed and statistically non-significant effects on muscle soreness (SMD = −0.06, 95% CI: [−0.32, 0.19], p = 0.63), strength (SMD = 0.27, 95% CI: [−0.14, 0.68], p = 0.19), performance (SMD = 0.18, 95% CI: [−0.11, 0.46], p = 0.22), flexibility (SMD = −0.06, 95% CI: [−0.31, 0.20], p = 0.67), and pain threshold (SMD = −0.02, 95% CI: [−0.41, 0.37], p = 0.93). Sensitivity analysis and Egger’s test indicated robust results and no publication bias.

**Conclusion:**

Post-exercise stretching, when used as a standalone recovery intervention, does not significantly improve soreness, strength, performance, flexibility, or pain threshold. While physiologically safe and practical, its effectiveness may be limited, warranting integration with multimodal recovery strategies in future applications.

**Systematic Review Registration:**

Identifier CRD420251113484.

## Introduction

Post-exercise stretching is a common intervention that is widely used in sports training, sports conditioning, and rehabilitation programs (Mašić et al., 2024). Its popularity stems primarily from the deep-seated belief among coaches, athletes, and rehabilitation professionals that stretching aids muscle recovery, reduces injury risk, and improves subsequent athletic performance (Herbert et al., 2011). These practices are often institutionalized in sports protocols, with many athletic teams and rehabilitation centers incorporating standardized stretching routines into post-exercise cooldown periods (Zvetkova et al., 2023; Takeuchi et al., 2019). Despite its widespread use in exercise regimens, the scientific community remains divided on its efficacy and potential physiological benefits (Behm et al., 2023; Opplert and Babault, 2018). This disagreement stems from inconsistent empirical research findings, which has led to controversy and ongoing debate among practitioners and researchers.

Several physiological mechanisms have been proposed to explain the purported benefits of post-exercise stretching. First, stretching is thought to reduce muscle stiffness by improving tendon compliance, potentially reducing the risk of muscle strain and improving exercise efficiency (Konrad et al., 2017). This mechanical response is believed to be mediated by viscoelastic changes in muscle-tendon units, resulting in temporary increases in range of motion (Takeuchi et al., 2023). Second, there are studies that suggest stretching can enhance local blood flow, increase nutrient and oxygen delivery facilitating the removal of metabolic byproducts such as lactic acid that accumulate during intense exercise (Kruse and Scheuermann, 2017). Third, stretching interventions are thought to alleviate delayed onset muscle soreness (DOMS), a common post-exercise phenomenon characterized by muscle discomfort, stiffness, and reduced exercise performance (Behm et al., 2021). Finally, it has been theorized that improved clearance of metabolic byproducts could accelerate tissue repair and recovery processes, potentially shortening the necessary recovery period between training sessions (Yan et al., 2020). In this context, stretching is often perceived as a low-cost, low-risk recovery modality suitable for a wide range of populations, from elite athletes to recreational exercisers (Ozsoy et al., 2024).

However, empirical evidence supporting these mechanisms remains variable and often conflicting. Numerous studies using randomized controlled trial (RCT) designs and different stretching protocols have investigated these claims, but results have often been inconsistent. Some studies have reported small reductions in perceived muscle soreness and stiffness following stretching, while others have failed to observe significant physiological or functional improvements (Warneke et al., 2024a; Herbert and Gabriel, 2002). Furthermore, studies examining key performance parameters such as muscle strength, power output, flexibility, endurance, and sprint speed have generally demonstrated minimal to no significant effects following stretching interventions (Warneke et al., 2022; Konrad et al., 2021; Warneke et al., 2024b).

In some cases, recent findings suggest that post-exercise stretching may have negative effects (Chaabene et al., 2019; McGrath et al., 2014). Transient decreases in muscle strength, power generation, and neuromuscular performance have been documented immediately following stretching training (Kallerud and Gleeson, 2013). These results raise an important question as to whether stretching training is appropriate immediately following exercise, especially in the context of subsequent competitive events or high-intensity training with a short recovery period (Thacker et al., 2004). This is particularly critical in sports with condensed schedules, where optimal recovery strategies are essential for maintaining peak performance. Despite these empirical observations, a gap remains between scientific evidence and practical application. Athletes and coaches continue to endorse stretching exercises, primarily based on anecdotal experience and subjectively perceived benefits rather than demonstrable physiological effects (Warneke et al., 2024c). This reliance on tradition and experiential knowledge underscores a broader issue in sports science: the challenge of translating empirical data into applied practice.

This persistent discrepancy has prompted multiple systematic reviews and meta-analyses aimed at reconciling the disparate findings. However, consensus remains elusive due to methodological heterogeneity, which is primarily attributed to differences in the types of stretching (e.g., static, dynamic, proprioceptive neuromuscular facilitation), duration and intensity of stretching interventions, targeted muscle groups, and different measures used to assess muscle recovery and performance (Afonso et al., 2024; Afonso et al., 2021a; Freitas et al., 2018). Furthermore, many meta-analyses group stretching interventions with broader recovery strategies, complicating direct comparisons and interpretations of stretching itself (Pooley et al., 2020; Sedano and Maroto-Izquierdo, 2025; Afonso et al., 2021b).

In summary, the ongoing controversy surrounding the efficacy of post-exercise stretching underscores the need for this meta-analysis. By systematically synthesizing high-quality evidence, this study aims to clarify whether post-exercise stretching facilitates muscle recovery and enhances subsequent performance, thereby providing a scientific basis for its application in training and rehabilitation practice.

## Methods

This study adhered to the preferred Reporting Items for Systematic Reviews and Meta-Analyses (PRISMA) guidelines and was registered in the PROSPERO database CRD420251113484 (Page et al., 2021).

### Eligibility criteria

Inclusion criteria were as follows: (Mašić et al., 2024) participants were healthy individuals with no history of disease; (Herbert et al., 2011) the study design was a randomized controlled trial (RCT), controlled clinical trial (CCT), or crossover trial; (Zvetkova et al., 2023) the experimental group received post-exercise relaxation primarily involving stretching, including static stretching, dynamic stretching, or proprioceptive neuromuscular facilitation (PNF), while the control group did not receive stretching-based relaxation, but all other conditions remained consistent between groups; (Takeuchi et al., 2019) outcome measures included at least one of the following: muscle soreness, muscle strength, flexibility, pain threshold or performance enhancement, with sufficient data to calculate the effect size (ES); and (Behm et al., 2023) full text of the article was available.

Exclusion criteria were as follows: (Mašić et al., 2024) studies that did not meet the inclusion criteria; (Herbert et al., 2011) studies lacking data necessary for effect size (ES) calculation; and (Zvetkova et al., 2023) studies without a control group or with a control group receiving active recovery interventions.

### Information sources

A comprehensive literature search was conducted across eight databases: Web of Science, pubMed, SPORTDiscus, ScienceDirect, Scopus, Cochrane Library, Embase, and proQuest. Notably, to broaden the scope of relevant literature, the search strategy extended beyond the initial protocol registered in PROSPERO. No restrictions were placed on language, publication type, or publication date. The final search was completed on 20 July 2025. In addition, supplementary sources such as Google Scholar and ResearchGate were reviewed, and the reference lists of relevant articles were manually screened to identify additional eligible studies.

### Search strategy

A researcher performed the database search using Boolean logic with the following search terms: (“post-exercise” OR “after exercise” OR “following exercise”) AND (stretching OR “static stretching” OR “dynamic stretching” OR “passive stretching” OR “active stretching” OR “range of motion” OR flexibility OR mobility) AND (“recovery” OR “muscle soreness” OR DOMS OR fatigue OR “muscle damage” OR “creatine kinase” OR CK OR “exercise recovery”) AND (“performance” OR strength OR power OR “jump height” OR “sprint” OR “explosive strength” OR “muscle force”). The detailed search strategy is provided in Supplementary Appendix A. Two independent reviewers imported all retrieved records into EndNote X9 for reference management and duplicate removal. Titles and abstracts were independently screened based on predefined inclusion criteria. All reviewers received standardized training before screening to ensure consistency. After the initial screening, full-text articles of potentially eligible studies were assessed against the inclusion criteria. Any discrepancies between the two reviewers were resolved through discussion with a third reviewer to reach consensus.

### Data extraction

Each study was coded based on the following variables: first author, participant characteristics (number, sex, age, and training experience), experimental group/control group treatment, stretched area, intervention time and intensity, and outcome measures (Table 1). Data extraction was performed independently by two reviewers. Discrepancies were resolved through discussion, and if necessary, a third reviewer was consulted to adjudicate unresolved conflicts. Although inter-rater reliability statistics were not calculated, all discrepancies were resolved through discussion. The study did not employ Covidence or similar software; therefore, reliability data could not be exported. Intervention methods in the experimental groups included static stretching, dynamic stretching, and proprioceptive neuromuscular facilitation (PNF). Outcome measures included pain scores, pain thresholds, strength, flexibility, and performance. If there were significant baseline differences between groups, the corresponding results were excluded from the analysis. For studies including two or more experimental groups, the sample size of the control group was proportionally divided according to Cochrane Handbook recommendations, so that each experimental group could be independently compared with a portion of the control group (Vesterinen et al., 2014). When outcomes were presented as bar graphs with error bars but without reporting exact means and standard deviations, the data were extracted using digitizing software by a designated reviewer (Vesterinen et al., 2014).

**TABLE 1:** Basic characteristics of included studies in the meta-analysis (n = 15). M + SD M = mean, SD = standard deviatio.

Study (year)	Subjects (sex)	Age	Sports experience	Experimental group/Control group treatment	Stretched area	Intervention frequency	Session duration	Intervention intensity	Outcome measures
Apostolopoulos et al., 2018	30 (30M)	25 ± 6	trained	static stretching/no stretching	quadriceps, hip flexors, hamstrings	1 time per day of 3 days	3 muscle groups * 3 times * 60 s	70%–80% maximum perceived stretch/30%–40% maximum perceived stretch	muscle soreness strength
Sohail et al., 2022	45 (Not reported)	G1: 30.47 ± 5.14 G2: 26.20 ± 5.55 G3: 27.80 ± 5.40	trained	G1: static stretching, G2: PNF stretching/no stretching	gastrocnemius, soleus	2 times per day of 5 days	G1: 10 times * 30 s G2: 15 times	mild discomfort/based on the maximum tolerated extension when the patient reaches the point of restricted range of motion	muscle soreness
Fakhro et al., 2020	96 (96M)	24.7 ± 4.1	trained	G1: static stretching, G2: dynamic stretching/no stretching	hamstrings	3 times per week of 4 weeks	3 times * 30 s	slight pain or discomfort/proactively swing until a stretching sensation is felt	Flexibility performance
Torres et al., 2013	28 (28M)	21.4 ± 1.9	untrained	static stretching/no stretching	hamstrings, quadriceps	4 times	10 times * 30 s	reasonable resistance or discomfort	muscle soreness strength flexibility
Study (year)	Subjects (sex)	Age	Sports experience	Experimental group/Control group treatment	Stretched area	Intervention frequency	Session duration	Intervention intensity	Outcome measures
Ozmen et al., 2017	48 (48F)	19.17–19.41	untrained	G1: static stretching, G2: PNF stretching/no stretching	hamstrings, quadriceps	1 time	G1: 5 times * 30 s G2: 10-s isometric contraction * 3times +10-s active contraction * 3 times	Until a subjective sensation of discomfort or tension in the hamstrings is felt	muscle soreness flexibility
Xie et al., 2018	48 (20M28F)	21.7 ± 1.40	untrained	G1: static stretching, G2: dynamic stretching/no stretching	gastrocnemius, soleus	2 times per day of 5 days	Not reported	stretch to the point of mild discomfort	muscle soreness flexibility pain threshold
Muanjai and Namsawang, 2015	26 (26M)	20.9 ± 1.1	untrained	static stretching/no stretching	quadriceps	1 time	5 times * 30 s * 2 groups	a noticeable stretching sensation or slight discomfort	muscle soreness
McGrath et al., 2014	57 (28M29F)	18–25	untrained	G1: static stretching, G2: PNF stretching/no stretching	hamstrings, guadriceps	1 time	Not reported	based on the maximum stretch sensation tolerable by the subject	muscle soreness flexibility
Study (year)	Subjects (sex)	Age	Sports experience	Experimental group/Control group treatment	Stretched area	Frequency	Session duration	Intervention intensity	Outcome measures
McGlynn et al., 1979a	24 (24M)	18–26	untrained	static stretching/no stretching	biceps	1 time per day of 5 days	15 min at a time	forearm hyperextension	muscle soreness
Wessel and Wan, 1994	10 (5M5F)	25.2 ± 3.36	untrained	static stretching/no stretching	lower limb muscles	1 time	Not reported	a distinct pulling sensation or slight discomfort	muscle soreness pain threshold
Kruse et al., 2013	11 (11F)	20.00 ± 1.55	trained	G1: static stretching, G2: dynamic stretching/no stretching	lower limb muscles	3 times	Not reported	mild discomfort/range of motion and full joint mobility	performance
Pooley et al., 2017	10 (10M)	16 ± 1	trained	static stretching/no stretching	gastrocnemius, hamstrings, quadriceps femoris, gluteals, hip flexors, adductor, abductors	1 time	2 times * 15 s * 7 groups	slight discomfort	muscle soreness performance
Study (year)	Subjects (sex)	Age	Sports experience	Experimental group/Control group treatment	Stretched area	Intervention frequency	Session duration	Intervention intensity	Outcome measures
West et al., 2014	12 (12M)	21.3 ± 2.3	trained	static stretching/no stretching	hamstrings, quadriceps, gluteals, ankle joints, etc.	1 time	12 min at a time	mild discomfort but not pain	performance
Mika et al., 2007	10 (10M)	24–38	untrained	static stretching/no stretching	quadriceps	1 time	5 min at a time	emphasise gentle, gradual stretching and avoid pain	strength
Johansson et al., 1999	10 (10F)	24 ± 3	trained	static stretching/no stretching	lower limb muscles	1 time	4 times * 20 s	a distinct sensation of stretching, without pain	muscle soreness pain threshold

### Study risk of bias assessment

According to the PROSPERO registration protocol, the risk of bias in included randomized controlled trials (RCTs) and controlled clinical trials (CCTs) was assessed using the Cochrane Risk of Bias 2.0 tool (RoB 2). For randomized crossover trials or pre-post crossover designs, the RoB 2 variant specific to crossover trials was applied (Vesterinen et al., 2014). The tool for assessing RCTs evaluates five domains of bias: (1) bias arising from the randomization process, (2) bias due to deviations from intended interventions, (3) bias due to missing outcome data, (4) bias in measurement of the outcome, and (5) bias in selection of the reported result. The crossover trial-specific variant includes all five domains above, with an additional domain: bias arising from period and carryover effects. Each domain contains several signaling questions. Based on the responses to these questions, the risk of bias is judged and assigned to one of three levels: “low risk of bias,” “some concerns,” or “high risk of bias” (Vesterinen et al., 2014). Assessments were independently conducted by two reviewers. Disagreements were resolved through discussion with a third reviewer until consensus was reached.

### Synthesis of results

As this review included both parallel-group and crossover trials, all outcome data were first converted into standardized mean difference (SMD) and standard error (SE). Meta-analyses were performed using Stata version 17.0, which was also used to generate forest plots and funnel plots. Sensitivity analyses and Egger’s test for and meta-regression analyses were conducted using Stata version 17.0. Meta-regression was performed with stretching type (static, dynamic, PNF) and training level (trained vs. untrained) as covariates to explore potential sources of heterogeneity. Given the expected clinical and methodological diversity among studies, a random-effects model was applied to analyze post-intervention data. Heterogeneity, defined as the variability in effect sizes across studies, was assessed using the chi-square test (p-value) and the I^2^ statistic. Heterogeneity was considered low if p > 0.1 and I^2^ < 40%. Due to the diversity of measurement units among included outcomes, SMD were used for pooled analysis. Statistical significance was defined as p < 0.05. The magnitude of SMD was interpreted as follows: trivial (<0.2), small (0.2–0.49), moderate (0.5–0.79), and large (≥0.8).

The certainty of evidence for each outcome was assessed using the GRADE approach. A Summary of Findings table was prepared (see Supplementary Appendix D).

## Results

### Study characteristics

**FIGURE 1:**
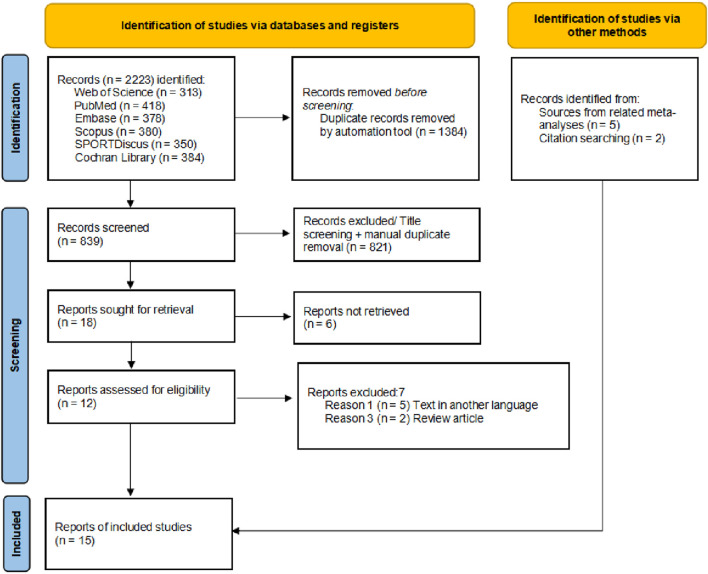
Flowchart of literature screening for systematic review and meta-analysis.

The flow diagram illustrates the complete process of the systematic literature search conducted for this study (Figure 1). A total of 2223 records were identified through database searching, and after sequential screening, eight articles (Wessel and Wan, 1994; Ozmen et al., 2017; Sohail et al., 2022; Torres et al., 2013; Pooley et al., 2017; Ap et al., 2018; Xie et al., 2018; Kruse et al., 2013) met the inclusion criteria. Seven additional eligible articles (McGrath et al., 2014; Wessel and Wan, 1994; McGlynn et al., 1979a; Mika et al., 2007; West et al., 2014; Muanjai and Namsawang, 2015; Johansson et al., 1999) was identified through other sources, resulting in 15 studies included in the final analysis (Table 1). After excluding intervention groups unrelated to the scope of this review, a total of 465 participants were included. Among the 15 included studies, 10 were parallel-group controlled trials (McGrath et al., 2014; Wessel and Wan, 1994; Ozmen et al., 2017; Sohail et al., 2022; Torres et al., 2013; Ap et al., 2018; Xie et al., 2018; McGlynn et al., 1979a; Fakhro et al., 2020; Muanjai and Namsawang, 2015), and 5 were crossover trials (Pooley et al., 2017; Kruse et al., 2013; Mika et al., 2007; West et al., 2014; Johansson et al., 1999). Eight studies included male participants only, three studies included female participants only, another three included both male and female participants, and one study did not report the sex distribution. Regarding age, one study involved minors, while the remaining 14 included adult participants. Seven studies reported that participants had a background in physical training or sports experience. In terms of intervention methods, most studies used static stretching; only three studies employed dynamic stretching, and three used PNF. The targeted muscle groups were almost exclusively located in the lower limbs. Regarding the duration and intensity of interventions, the majority of studies implemented acute interventions, comprising single sessions or no more than three sessions. Fourteen studies reported the frequency or duration of single stretching sessions. Regarding outcome measures, 11 studies reported muscle soreness scores, 3 studies assessed strength-related outcomes, 4 studies examined performance-related outcomes, 5 studies measured flexibility, and 3 studies reported pain threshold data. In addition, data from 3 studies were estimated from figures using digitizing software (Wessel and Wan, 1994; Johansson et al., 1999).

**FIGURE 2:**
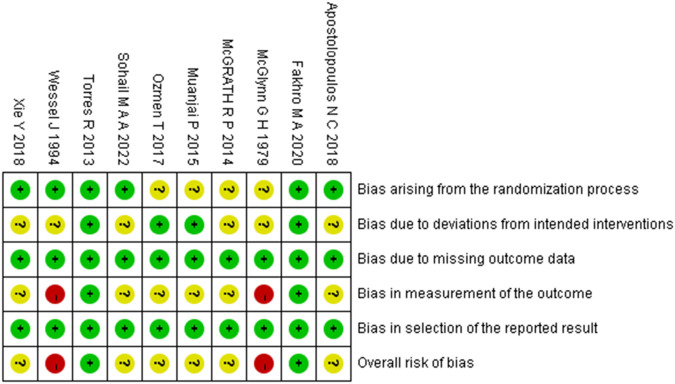
Summary of risk-of-bias judgments across randomised controlled trial using the RoB 2 tool. Each domain is evaluated as low risk (green), some concerns (yellow), or high risk (red).

**FIGURE 3:**
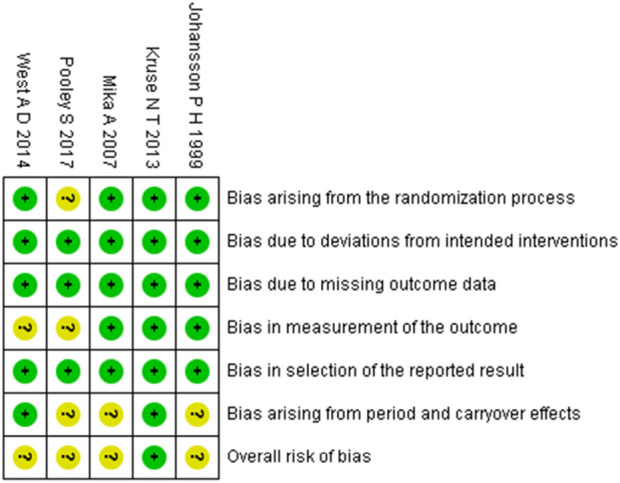
Summary of risk-of-bias judgments across randomised crossover trial using the RoB 2 tool. Each domain is evaluated as low risk (green), some concerns (yellow), or high risk (red).

The methodological quality of the included studies was assessed using the RoB 2 tool. The risk of bias assessments for randomized controlled trials and randomized crossover trials are presented in Figures 2, 3, respectively. Detailed assessment criteria and domain-specific proportions are provided in Supplementary Appendices B, C. Among the 15 included studies, the overall methodological quality was judged as moderate, with several domains rated as having “some concerns” or “high risk of bias.” Specifically, most studies were rated as having low risk of bias in the domains related to missing outcome data and selective reporting. However, concerns frequently arose in the domains of the randomization process, deviations from intended interventions, and measurement of outcomes. Two studies were judged to have an overall high risk of bias due to being rated as high risk in one or more domains (Wessel and Wan, 1994; McGlynn et al., 1979b). In total, 10 studies were rated as having “some concerns,” and 3 studies were rated as having an overall low risk of bias. These findings underscore the need for more rigorous study designs in future research, including clear reporting of randomization procedures and strict implementation of blinding methods.

### Holistic analysis

**FIGURE 4:**
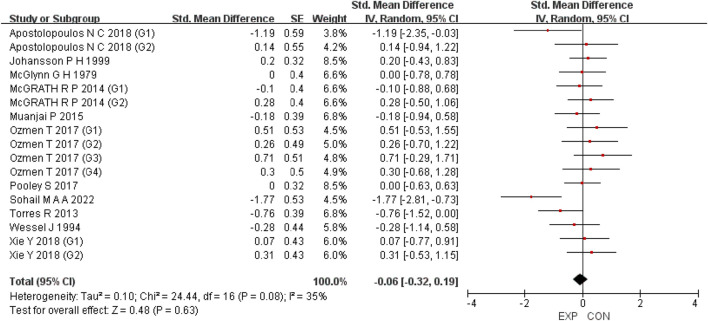
Forest plot showing the overall effect of stretching on muscle soreness. The pooled analysis indicated no significant reduction in soreness, with a trivial effect size.

**FIGURE 5:**
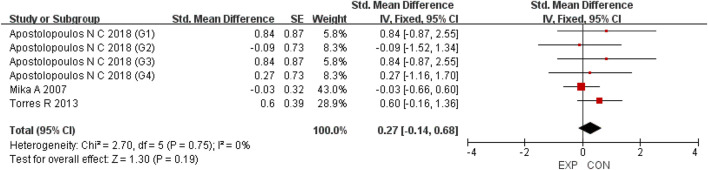
Forest plot showing the overall effect of stretching on strength. A small but non-significant trend toward improved strength was observed.

**FIGURE 6:**
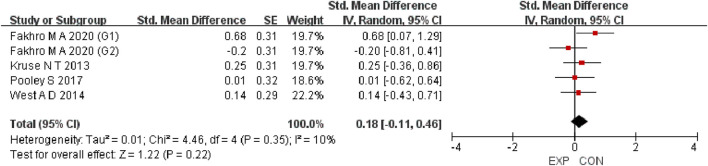
Forest plot showing the overall effect of stretching on performance. Results showed no meaningful enhancement in jump, sprint, or other performance measures.

**FIGURE 7:**
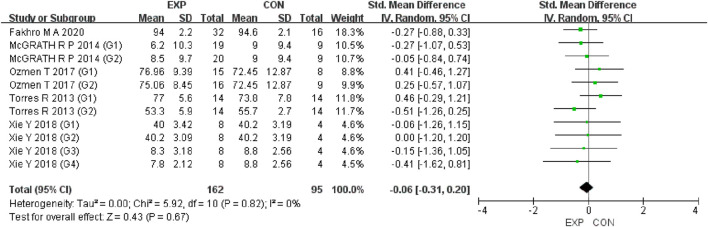
Forest plot showing the overall effect of stretching on flexibility. The analysis did not demonstrate short-term flexibility gains following post-exercise stretching.

**FIGURE 8:**
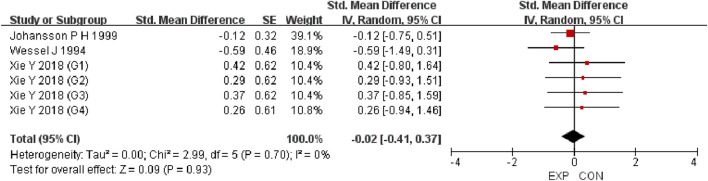
Forest plot showing the overall effect of stretching on pain threshold. Findings suggest stretching did not alter pain sensitivity compared with controls.

A total of 15 studies were included to evaluate the overall effects of post-exercise stretching on muscle soreness, muscle strength, performance, flexibility, and pain threshold. Using a random-effects model, the results showed that post-exercise stretching had a pooled SMD of −0.06 for muscle soreness (95% CI: [−0.32, 0.19], p = 0.63; Chi^2^ = 24.44, p = 0.08; I^2^ = 35%) (Figure 4), −0.02 for pain threshold (95% CI: [−0.41, 0.37], p = 0.93; Chi^2^ = 2.99, p = 0.70; I^2^ = 0%) (Figure 5), 0.18 for performance (95% CI: [−0.11, 0.46], p = 0.22; Chi^2^ = 4.46, p = 0.35; I^2^ = 10%) (Figure 6), 0.27 for strength (95% CI: [−0.14, 0.68], p = 0.19; Chi^2^ = 2.70, p = 0.75; I^2^ = 0%) (Figure 7), and −0.06 for flexibility (95% CI: [−0.31, 0.20], p = 0.67; Chi^2^ = 5.92, p = 0.82; I^2^ = 0%) (Figure 8). These findings suggest that post-exercise stretching has trivial and statistically non-significant effects on muscle soreness, flexibility, and pain threshold (SMD <0.2), and small but non-significant effects on strength and performance (0.2 ≤ SMD <0.5). Heterogeneity across outcomes was low (all I^2^ ≤ 35%), supporting the robustness of the results.

To assess potential publication bias, funnel plots were visually inspected and Egger’s tests were performed (SupplementaryAppendix C). The funnel plots appeared symmetrical, and the Egger’s tests revealed no significant evidence of publication bias (all p > 0.05), suggesting the absence of substantial reporting bias among the included studies. Although small-study effects cannot be entirely ruled out, the visual symmetry of the funnel plots provides additional reassurance that such effects are unlikely to have materially influenced the overall findings.

### Subgroup analyses by stretching frequency

**FIGURE 9:**
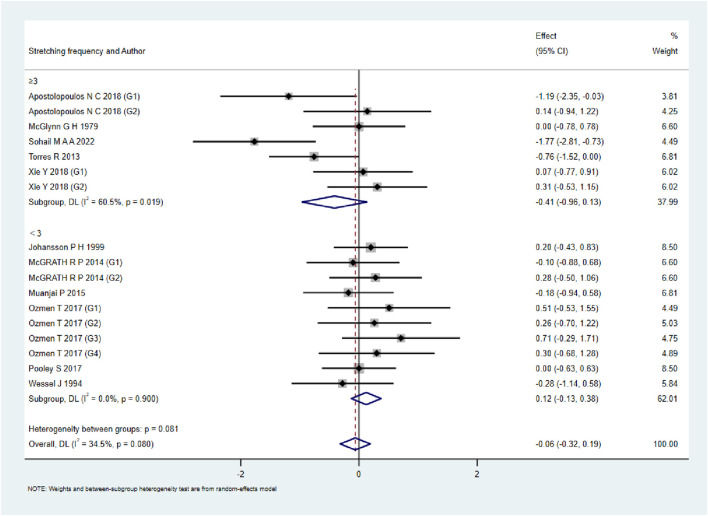
Subgroup analysis of stretching frequency (≥3 vs. <3 sessions) for muscle soreness.

**FIGURE 10:**
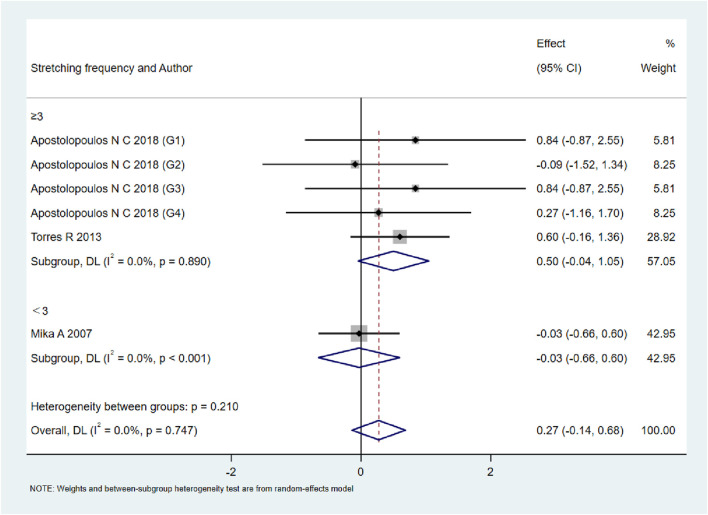
Subgroup analysis of stretching frequency (≥3 vs. <3 sessions) for strength.

**FIGURE 11:**
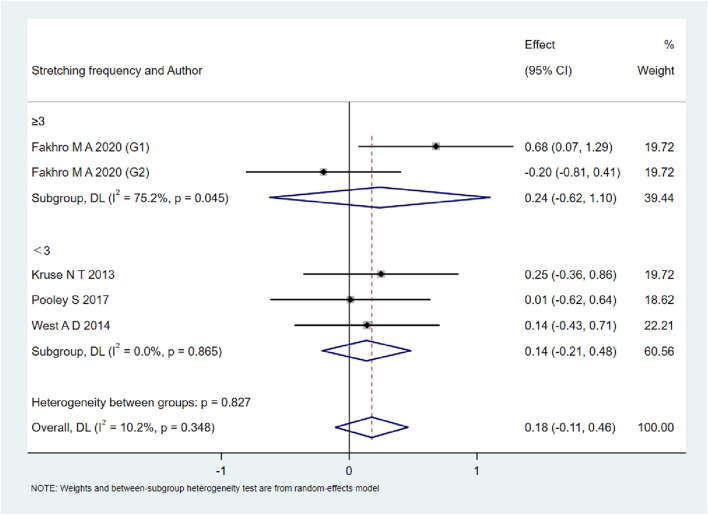
Subgroup analysis of stretching frequency (≥3 vs. <3 sessions) for performance.

**FIGURE 12:**
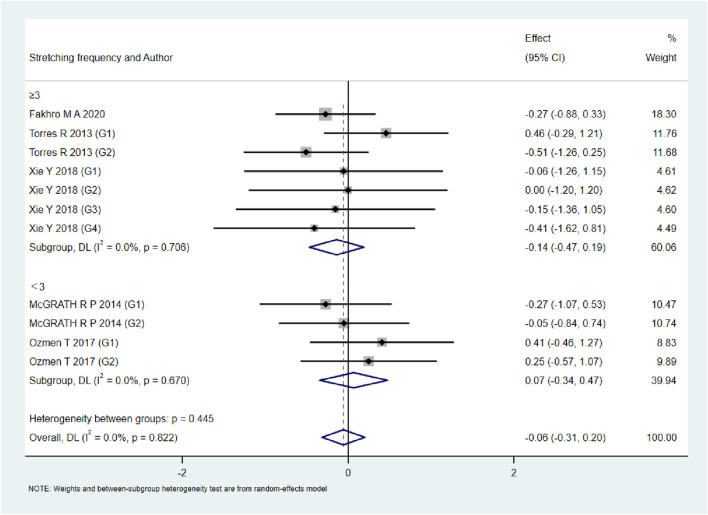
Subgroup analysis of stretching frequency (≥3 vs. <3 sessions) for flexibility.

**FIGURE 13:**
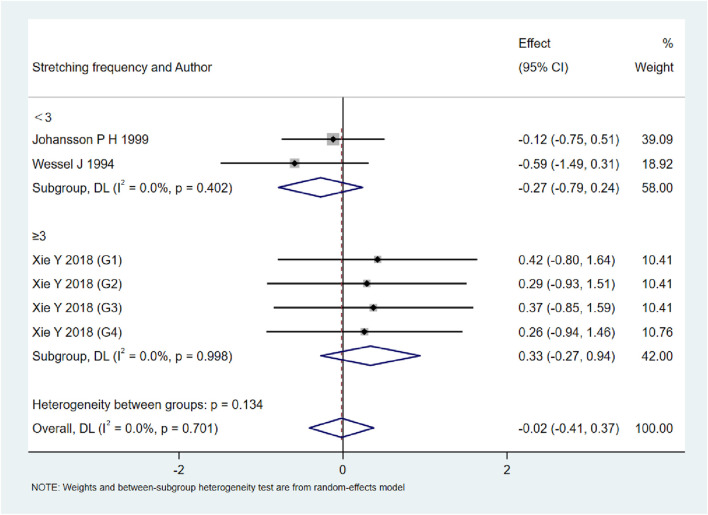
Subgroup analysis of stretching frequency (≥3 vs. <3 sessions) for pain threshold.

As shown in Figures 9–13, when studies were stratified according to stretching frequency (≥3 sessions vs. <3 sessions), no significant between-subgroup differences were observed for any outcome. For flexibility, the pooled effect was −0.14 (95% CI −0.47 to 0.19; I^2^ = 0.0%) in the ≥3 sessions subgroup and 0.07 (95% CI −0.34 to 0.47; I^2^ = 0.0%) in the <3 sessions subgroup, with no significant difference between them (Q_between p = 0.445). For muscle soreness, the ≥3 sessions subgroup showed an effect of −0.41 (95% CI −0.96 to 0.13; I^2^ = 60.5%), while the <3 sessions subgroup showed 0.12 (95% CI −0.13 to 0.38; I^2^ = 0.0%); the difference was not significant (Q_between p = 0.081). For pain threshold, the pooled effect was 0.33 (95% CI −0.27 to 0.94; I^2^ = 0.0%) in the ≥3 sessions subgroup and −0.27 (95% CI −0.79 to 0.24; I^2^ = 0.0%) in the <3 sessions subgroup (Q_between p = 0.134). For athletic performance, the effect was 0.24 (95% CI −0.62 to 1.10; I^2^ = 75.2%) in the ≥3 sessions subgroup and 0.14 (95% CI −0.21 to 0.48; I^2^ = 0.0%) in the <3 sessions subgroup (Q_between p = 0.827). For strength/torque, the pooled effect was 0.50 (95% CI −0.04 to 1.05; I^2^ = 0.0%) in the ≥3 sessions subgroup and −0.03 (95% CI −0.66 to 0.60; I^2^ = 0.0%) in the <3 sessions subgroup (Q_between p = 0.210). Collectively, these findings indicate that increasing stretching frequency to three sessions or more did not yield superior effects compared with lower-frequency protocols.

### Sensitivity analysis

A leave-one-out sensitivity analysis was conducted to assess the robustness of the findings (Supplementary Appendix C). The results showed that no individual study exerted a disproportionate influence on the overall estimates. This indicates that the observed effects of post-exercise stretching on muscle soreness, strength, performance, flexibility, and pain threshold were not driven by any single study, and the overall findings remained stable when each study was excluded in turn. According to the GRADE evaluation, the certainty of evidence for all outcomes was rated as low, primarily due to risk of bias and imprecision (Supplementary Appendix D).

Additionally, when the two studies judged to be at overall high risk of bias were excluded, the pooled results remained unchanged (Supplementary Appendix E).

### Meta-regression analysis

To further explore potential sources of heterogeneity, meta-regression analyses were performed using stretching type (static/dynamic/PNF) and training level (trained/untrained) as covariates.

For muscle soreness, neither stretching type (β = 0.17, p = 0.387) nor training level (β = 0.31, p = 0.336) was significantly associated with the effect size, with residual heterogeneity remaining moderate (I^2^ = 35%). For pain threshold, both stretching type (β = 0.53, p = 0.400) and training level (β = −0.01, p = 0.978) were not significant predictors (I^2^ = 0%). For performance, neither stretching type (β = −0.47, p = 0.268) nor training level (omitted due to collinearity) significantly explained the variation (I^2^ = 0%). For strength outcomes, training level (β = −0.17, p = 0.735) was not a significant moderator, with stretching type omitted due to collinearity. For flexibility, neither stretching type (β = 0.02, p = 0.927) nor training level (β = 0.25, p = 0.494) showed a moderating effect (I^2^ = 0%).

Overall, no covariates significantly explained the heterogeneity across outcomes, suggesting that stretching type and training level did not influence the effectiveness of post-exercise stretching.

## Discussion

This systematic review and meta-analysis aimed to evaluate the effects of post-exercise stretching on muscle recovery and athletic performance. Despite its widespread application and long-standing theoretical support, the pooled evidence using random-effects models with Hedges’ g correction did not demonstrate statistically significant improvements in any of the key outcome domains examined, including pain perception, pain threshold, athletic performance, muscle strength, and flexibility. Sensitivity analyses, including the exclusion of high risk-of-bias studies, confirmed the robustness of these findings. Collectively, the results suggest that, when applied as a stand-alone intervention, stretching may have limited physiological efficacy in facilitating recovery, and its clinical impact may be overestimated.

Stretching has traditionally been advocated as a recovery tool based on its proposed physiological mechanisms, including enhanced circulation, accelerated removal of metabolic byproducts, reduction in muscle stiffness, and modulation of neuromuscular tension (Daneshjoo et al., 2024). While such mechanisms are biologically plausible, our findings indicate that these changes—if present—do not consistently translate into measurable functional recovery (Warneke et al., 2024c). For instance, in the context of pain-related outcomes, although isolated studies have reported transient reductions in perceived soreness following stretching, the overall pooled data do not support a robust analgesic effect (Støve et al., 2025; Støve et al., 2024a; Støve et al., 2024b). The complex and multifactorial nature of DOMS, which involves peripheral inflammation, microtrauma, and central sensitization, may limit the potential of stretching to significantly modulate pain perception (Mizumura and Taguchi, 2024). Since stretching mainly works on muscles and joints rather than directly on the body’s pain pathways, its ability to reduce pain is quite limited and not strong enough to have real therapeutic value (Konrad et al., 2025; Anderson et al., 2024).

Pain threshold data further reinforce this conclusion, with most studies showing minimal or inconsistent changes following stretching interventions (Støve et al., 2025; Støve et al., 2024a; Fernández-del Rivero et al., 2025). Unlike subjective pain ratings, pain threshold assessments are considered somewhat more objective as they can be quantified with greater precision; however, they are not immune to placebo or expectancy effects (Locher et al., 2017). However, they may still be influenced by placebo or expectancy effects and should not be regarded as entirely immune to such biases. The absence of change in this parameter suggests that stretching may have limited influence on nociceptive modulation, and its role in managing exercise-induced pain should be interpreted with caution, particularly when applied as a stand-alone strategy (Zedka et al., 1999). Therefore, its utility as a recovery strategy for managing exercise-induced pain remains questionable, particularly when used in isolation.

In terms of athletic performance, the findings were similarly inconclusive. Stretching has often been incorporated into post-exercise routines under the assumption that it may enhance subsequent performance through increased muscle compliance, reduced stiffness, or improved neuromuscular coordination (Konrad et al., 2024). However, the current synthesis found no consistent improvement across performance metrics such as jump height, sprint time, or balance. In fact, some studies have suggested that stretching—especially static stretching performed for extended durations—can acutely reduce force output and neuromuscular activation, likely through alterations in the length–tension relationship of muscles and decreased motor unit recruitment (Trajano et al., 2017). It should be noted that different stretching modalities may elicit distinct effects on performance. For instance, static stretching has been associated with transient decreases in explosive strength, whereas dynamic stretching may acutely enhance performance through increased neuromuscular activation. By combining all stretching modalities in a pooled analysis, such modality-specific effects may have been masked (Trajano et al., 2017). This may be particularly relevant in the context of explosive or strength-based activities, where maximal voluntary contraction and rapid force generation are essential (Del Vecchio et al., 2019). The heterogeneity in performance assessment tools used across studies, combined with variability in subject populations and stretching protocols, further limits the generalizability of any observed effects (Maffiuletti et al., 2016).

Muscle strength, a key component of post-exercise recovery, was also unaffected by stretching in our analysis. While some hypothesize that stretching may support the restoration of contractile function by relieving residual tension or improving muscle extensibility, current data do not substantiate these claims (Harvey et al., 2017). Moreover, from a practical standpoint, it may be unrealistic to expect strength improvements to occur immediately after stretching; such recovery processes typically require 24–48 h in combination with adequate rest and nutrition. Strength outcomes—including isometric torque, concentric force, and rate of force development—remained largely unchanged across studies. This could be attributed to the fact that the mechanical and neural impairments induced by fatiguing exercise are unlikely to be reversed by passive interventions alone (Amiri and Zemková, 2025). While static stretching can sometimes be applied in a relatively passive manner (e.g., with external support), dynamic and PNF stretching typically require active muscular engagement, which makes them only partially “passive” in nature. Restoring muscular strength likely requires more targeted strategies that engage active remodeling and neuromuscular retraining, rather than relying solely on passive mechanical inputs.

One of the more surprising findings was the lack of significant improvement in flexibility, traditionally viewed as the most direct target of stretching. While flexibility gains are well-documented in long-term training programs, our analysis focused on short-term post-exercise interventions, which may not provide sufficient duration or intensity to elicit structural adaptations. Enhancing flexibility typically requires sustained, repetitive loading of musculotendinous units to promote viscoelastic remodeling (Oba et al., 2021). The included studies largely implemented brief stretching sessions with limited frequency, which may explain the absence of notable improvements. Moreover, measurement techniques for flexibility varied widely across studies—ranging from joint angle assessments to sit-and-reach tests—further complicating direct comparisons.

Several studies attempted to explore the underlying physiological changes induced by stretching, including alterations in inflammatory markers (e.g., IL-6, IL-10, TNF-α), changes in muscle architecture, and perfusion-related outcomes. However, these findings were sporadic and inconsistent. Even when biological changes were observed, they did not consistently align with improvements in functional recovery, underscoring the disconnect between mechanistic hypotheses and clinical effectiveness. Furthermore, the methodological quality and consistency of the included studies were highly variable. Many studies employed different stretching modalities (e.g., static, dynamic, PNF), with wide variation in duration, frequency, and supervision. In some cases, subgroups from the same trial population were repeatedly analyzed across different outcomes, which may have introduced redundancy and reduced the precision of pooled estimates.

These limitations highlight the need for caution when interpreting the efficacy of stretching. It is likely that the effectiveness of stretching depends on a range of contextual factors, including the timing of the intervention, the population being studied (e.g., trained athletes vs. recreational individuals), the specific recovery outcomes being targeted, and the concurrent use of other recovery strategies (Konrad et al., 2024). The lack of standardized intervention protocols makes it difficult to determine the optimal conditions under which stretching may be beneficial.

Based on the current evidence, post-exercise stretching should not be relied upon as a primary recovery strategy, as it does not significantly reduce soreness, improve strength, performance, flexibility, or pain threshold. Nevertheless, stretching may still serve useful roles in practice (Afonso et al., 2021b). For athletes and coaches in flexibility-demanding sports such as gymnastics, martial arts, or figure skating, stretching remains essential for maintaining joint range of motion and reducing injury risk. For clinicians working in rehabilitation settings, passive stretching can be applied during early recovery phases to preserve mobility and prevent contracture when active motion is limited (Prabhu et al., 2013). More broadly, stretching may offer psychological benefits, including reduced perceived fatigue and enhanced body awareness, which can support motivation and adherence. Thus, stretching is best recommended as a complementary, low-cost, and low-risk addition to multimodal recovery or rehabilitation programs, rather than as a stand-alone intervention.

Despite the absence of statistically significant effects across primary outcomes, stretching may still hold practical value in specific contexts. For sports that demand high flexibility—such as gymnastics, martial arts, or figure skating—stretching remains essential for maintaining performance readiness and preventing injury (Zvetkova et al., 2023). Additionally, in early rehabilitation settings, where active motion may be contraindicated, passive stretching can help preserve joint mobility and prevent contracture (McHugh and Cosgrave, 2009). Psychological benefits, such as reduced perceived fatigue, increased body awareness, and a subjective sense of recovery, although difficult to quantify, may also contribute to adherence and recovery motivation (McHugh and Cosgrave, 2009).

Importantly, stretching is often described as a low-resource recovery modality that can be performed with minimal equipment and environmental requirements. However, its physiological intensity can vary widely depending on the protocol, ranging from gentle, low-load stretching to higher-intensity regimens approaching the limits of individual tolerance (Behm et al., 2016). Compared with other recovery methods such as aerobic exercise, cryotherapy, or massage, stretching requires no specialized equipment, minimal space, and can be implemented almost anywhere (Behm et al., 2016). These attributes render it particularly practical during periods of travel, fatigue accumulation, or in environments where recovery resources are limited. Although static stretching alone may not yield significant physiological recovery benefits, its accessibility and negligible risk profile make it a viable complementary method within broader recovery programs.

Future research should explore the role of stretching within multimodal recovery frameworks, examining its potential synergistic effects when combined with other interventions. Standardizing stretching protocols—including duration, frequency, and intensity—and clarifying the specific outcomes it can reliably influence will be essential for developing evidence-based guidelines. Investigations should also consider stratifying participants by baseline flexibility, training level, and recovery needs to better personalize intervention strategies.

In summary, this meta-analysis found no compelling evidence that post-exercise stretching, when used alone, significantly improves pain, performance, strength, or flexibility. While its proposed physiological mechanisms remain plausible, their clinical relevance appears limited under typical implementation conditions. Nevertheless, due to its safety, accessibility, and low resource demands, stretching retains practical value in certain populations and contexts. Rather than serving as a primary recovery intervention, stretching may be best utilized as a complementary component within integrated recovery systems tailored to individual needs and specific performance goals.

### Future research

Although this meta-analysis found no significant benefits of post-exercise stretching on pain, strength, flexibility, or performance, several research gaps remain that merit further investigation. Future studies should prioritize the following directions: (1) participant stratification—explore how individual factors such as baseline flexibility, athletic level, and recovery demand influence the effectiveness of stretching. Heterogeneity in population characteristics may obscure potential subgroup-specific benefits. (2) protocol standardization—establish optimal stretching modalities, including type (e.g., static, dynamic, PNF), duration, frequency, and timing relative to exercise. A consistent framework is essential for drawing generalizable conclusions. (3) Multimodal recovery integration—examine the synergistic effects of stretching when combined with other recovery strategies (e.g., cold therapy, active recovery), especially under high-performance conditions. (4) Mechanistic research—investigate the neurophysiological and biochemical pathways underlying stretching’s effects, including its influence on inflammatory markers, muscle perfusion, and neuromuscular activation. Addressing these areas will help clarify the contextual utility of stretching and guide evidence-based recovery programming.

### Limitations

This meta-analysis has several limitations that should be acknowledged. Although we restricted our inclusion criteria to post-exercise stretching interventions, the specific protocols varied in stretching type, duration, and timing, which reflects real-world diversity but may have introduced minor variability in outcomes. Additionally, the focus on objective indicators such as pain, strength, and flexibility meant that potentially meaningful subjective experiences—like perceived recovery or psychological readiness—could not be evaluated due to inconsistent reporting across studies. Most participants were healthy young adults, which limits the generalizability of findings to older individuals, elite athletes, or clinical populations with distinct recovery demands. Although most included studies investigated acute, short-term interventions, seven of the fifteen studies implemented repeated sessions beyond a single bout. However, the duration and frequency were still relatively limited, and long-term adaptations remain insufficiently explored. Lastly, some variation existed in the measurement tools used across studies—for instance, different flexibility tests—which, although addressed through standardized effect size calculations, may still contribute to subtle differences in effect estimates.

## Conclusion

In conclusion, this systematic review and meta-analysis found no compelling evidence that post-exercise stretching, when implemented as a standalone intervention, produces statistically significant improvements in muscle soreness, strength, performance, flexibility, or pain threshold. While the physiological rationale for stretching remains plausible, its actual effects on recovery-related outcomes appear minimal under typical application conditions. Nonetheless, stretching maintains practical value due to its simplicity, accessibility, and low risk, particularly as a complementary element within broader, multimodal recovery strategies. Future research should focus on protocol standardization, individualized application, and integration with other recovery methods to better elucidate the contexts in which stretching may contribute meaningfully to post-exercise recovery.

## Data Availability

The original contributions presented in the study are included in the article/Supplementary Material, further inquiries can be directed to the corresponding author.

## Abbreviations

CCT, Controlled Clinical Trial; CI, Confidence Interval; CK, Creatine Kinase; DOMS, Delayed Onset Muscle Soreness; ES, Effect Size; IL-6, Interleukin-6; IL-10, Interleukin-10; MD, Mean Difference; pNF, proprioceptive Neuromuscular Facilitation; pRISMA, preferred Reporting Items for Systematic Reviews and Meta-Analyses; pROSpERO, International prospective Register of Systematic Reviews; RCT, Randomized Controlled Trial; RoB 2, Cochrane Risk of Bias 2.0 Tool; SE, Standard Error; SMD, Standardized Mean Difference; TNF-α, Tumor Necrosis Factor-alpha.

## References

[B1] Afonso J. Ramirez-Campillo R. Moscão J. Rocha T. Zacca R. Martins A. (2021a). Strength training versus stretching for improving range of motion: a systematic review and meta-analysis. Healthcare 9 (4), 427. 10.3390/healthcare9040427 33917036 PMC8067745

[B2] Afonso J. Clemente F. M. Nakamura F. Y. Morouço P. Sarmento H. Inman R. A. (2021b). The effectiveness of post-exercise stretching in short-term and delayed recovery of strength, range of motion and delayed onset muscle soreness: a systematic review and meta-analysis of randomized controlled trials. Front. Physiol. 12. 10.3389/fphys.2021.677581 34025459 PMC8133317

[B3] Afonso J. Andrade R. Rocha-Rodrigues S. Nakamura F. Y. Sarmento H. Freitas S. R. (2024). What we do not know about stretching in healthy athletes: a scoping review with evidence gap map from 300 trials. Sports Med. 54 (6), 1517–1551. 10.1007/s40279-024-02002-7 38457105 PMC11239752

[B4] Amiri B. Zemková E. (2025). Trunk stability and breathing exercises superior to foam rolling for restoring postural stability after core muscle fatigue in sedentary employees. Sci. Rep. 15 (1), 13909. 10.1038/s41598-025-98284-6 40263381 PMC12015519

[B5] Anderson A. W. Soncini A. Lyons K. Hanney W. J. (2024). The effect of myofascial stretching on mechanical nociception and contributing neural mechanisms. NeuroSci 5 (2), 158–168. 10.3390/neurosci5020011 39483492 PMC11493203

[B6] Apostolopoulos N. C. Lahart I. M. Plyley M. J. Taunton J. Nevill A. M. Koutedakis Y. (2018). The effects of different passive static stretching intensities on recovery from unaccustomed eccentric exercise – a randomized controlled trial. Appl. Physiol. Nutr. Metab. 43 (8), 806–815. 10.1139/apnm-2017-0841 29529387

[B7] Behm D. G. Blazevich A. J. Kay A. D. McHugh M. (2016). Acute effects of muscle stretching on physical performance, range of motion, and injury incidence in healthy active individuals: a systematic review. Appl. Physiol. Nutr. Metab. 41 (1), 1–11. 10.1139/apnm-2015-0235 26642915

[B8] Behm D. G. Kay A. D. Trajano G. S. Alizadeh S. Blazevich A. J. (2021). Effects of acute and chronic stretching on pain control. J. Clin. Exerc Physiol. 10 (4), 150–159. 10.31189/2165-6193-10.4.150

[B9] Behm D. G. Alizadeh S. Daneshjoo A. Konrad A. (2023). Potential effects of dynamic stretching on injury incidence of athletes: a narrative review of risk factors. Sports Med. 53 (7), 1359–1373. 10.1007/s40279-023-01847-8 37162736 PMC10289929

[B10] Chaabene H. Behm D. G. Negra Y. Granacher U. (2019). Acute effects of static stretching on muscle strength and power: an attempt to clarify previous caveats. Front. Physiol. 10, 1468. 10.3389/fphys.2019.01468 31849713 PMC6895680

[B11] Daneshjoo A. Hosseini E. Heshmati S. Sahebozamani M. Behm D. G. (2024). Effects of slow dynamic, fast dynamic, and static stretching on recovery of performance, range of motion, balance, and joint position sense in healthy adults. BMC Sports Sci. Med. Rehabil. 16 (1), 167. 10.1186/s13102-024-00841-5 39123262 PMC11312939

[B12] Del Vecchio A. Negro F. Holobar A. Casolo A. Folland J. P. Felici F. (2019). You are as fast as your motor neurons: speed of recruitment and maximal discharge of motor neurons determine the maximal rate of force development in humans. J. Physiol. 597 (9), 2445–2456. 10.1113/JP277396 30768687 PMC6487919

[B13] Fakhro M. A. Chahine H. Srour H. Hijazi K. (2020). Effect of deep transverse friction massage *vs* stretching on football players’ performance. World J. Orthop. 11 (1), 47–56. 10.5312/wjo.v11.i1.47 31966969 PMC6960298

[B14] Fernández-del Rivero C. García-Gil P. Mínguez-Cruz J. Pecos-Martín D. Fernández-Carnero S. Achalandabaso-Ochoa A. (2025). Effects of neural load on hamstring stretching upon flexibility, maximum isometric strength, and tibial nerve pressure pain threshold in healthy subjects: a randomized clinical trial. Appl. Sci. 15 (2), 683. 10.3390/app15020683

[B15] Freitas S. R. Mendes B. Le Sant G. Andrade R. J. Nordez A. Milanovic Z. (2018). Can chronic stretching change the muscle‐tendon mechanical properties? A review. Scand. J. Med. Sci. Sports 28 (3), 794–806. 10.1111/sms.12957 28801950

[B16] Harvey L. A. Katalinic O. M. Herbert R. D. Moseley A. M. Lannin N. A. Schurr K. (2017). Stretch for the treatment and prevention of contractures. Cochrane Database Syst. Rev. 1 (2), CD007455. Available online at: http://doi.wiley.com/10.1002/14651858.CD007455.pub3. 28146605 10.1002/14651858.CD007455.pub3 PMC6464268

[B17] Herbert R. D. Gabriel M. (2002). Effects of stretching before and after exercising on muscle soreness and risk of injury: systematic review. BMJ 325 (7362), 468. 10.1136/bmj.325.7362.468 12202327 PMC119442

[B18] Herbert R. D. De Noronha M. Kamper S. J. (2011). Stretching to prevent or reduce muscle soreness after exercise. Cochrane Database Syst. Rev., CD004577. Available online at: https://doi.wiley.com/10.1002/14651858.CD004577.pub3. 21735398 10.1002/14651858.CD004577.pub3

[B19] Johansson P. H. Lindström L. Sundelin G. Lindström B. (1999). The effects of preexercise stretching on muscular soreness, tenderness and force loss following heavy eccentric exercise. Scand. J. Med. Sci. Sports 9 (4), 219–225. 10.1111/j.1600-0838.1999.tb00237.x 10407930

[B20] Kallerud H. Gleeson N. (2013). Effects of stretching on performances involving stretch-shortening cycles. Sports Med. 43 (8), 733–750. 10.1007/s40279-013-0053-x 23681447

[B21] Konrad A. Stafilidis S. Tilp M. (2017). Effects of acute static, ballistic, and PNF stretching exercise on the muscle and tendon tissue properties. Scand. J. Med. Sci. Sports 27 (10), 1070–1080. 10.1111/sms.12725 27367916 PMC5479471

[B22] Konrad A. Močnik R. Nakamura M. Sudi K. Tilp M. (2021). The impact of a single stretching session on running performance and running economy: a scoping review. Front. Physiol. 11, 630282. 10.3389/fphys.2020.630282 33551850 PMC7857312

[B23] Konrad A. Alizadeh S. Daneshjoo A. Anvar S. H. Graham A. Zahiri A. (2024). Chronic effects of stretching on range of motion with consideration of potential moderating variables: a systematic review with meta-analysis. J. Sport Health Sci. 13 (2), 186–194. 10.1016/j.jshs.2023.06.002 37301370 PMC10980866

[B24] Konrad A. Nakamura M. Sardroodian M. Aboozari N. Anvar S. H. Behm D. G. (2025). The effects of chronic stretch training on musculoskeletal pain. Eur. J. Appl. Physiol. 125, 2037–2048. 10.1007/s00421-025-05747-9 40059246 PMC12354564

[B25] Kruse N. T. Scheuermann B. W. (2017). Cardiovascular responses to skeletal muscle stretching: “stretching” the truth or a new exercise paradigm for cardiovascular medicine? Sports Med. 47 (12), 2507–2520. 10.1007/s40279-017-0768-1 28780647

[B26] Kruse N. T. Barr M. W. Gilders R. M. Kushnick M. R. Rana S. R. (2013). Using a practical approach for determining the Most effective stretching strategy in female college division I volleyball players. J. Strength Cond. Res. 27 (11), 3060–3067. 10.1519/JSC.0b013e31828bf2b6 23442280

[B27] Locher C. Frey Nascimento A. Kirsch I. Kossowsky J. Meyer A. Gaab J. (2017). Is the rationale more important than deception? A randomized controlled trial of open-label placebo analgesia. Pain 158 (12), 2320–2328. 10.1097/j.pain.0000000000001012 28708766

[B28] Maffiuletti N. A. Aagaard P. Blazevich A. J. Folland J. Tillin N. Duchateau J. (2016). Rate of force development: physiological and methodological considerations. Eur. J. Appl. Physiol. 116 (6), 1091–1116. 10.1007/s00421-016-3346-6 26941023 PMC4875063

[B29] Mašić S. Čaušević D. Čović N. Spicer S. Doder I. (2024). The benefits of static stretching on health: a systematic review. J. Kinesiol Exerc Sci. 34 (105), 1–7. 10.5604/01.3001.0054.2941

[B30] McGlynn G. H. Laughlin N. T. Rowe V. (1979a). Effect of electromyographic feedback and static stretching on artificially induced muscle soreness. Am. J. Phys. Med. 58 (3), 139–148. 453340

[B31] McGlynn G. H. Laughlin N. T. Rowe V. (1979b). Effect of electromyographic feedback and static stretching on artificially induced muscle soreness. Am. J. Phys. Med. 58 (3), 139–148. 453340

[B32] McGrath R. P. Whitehead J. R. Caine D. J. (2014). The effects of proprioceptive neuromuscular facilitation stretching on post-exercise delayed onset muscle soreness in young adults. Int. J. Exerc Sci. 7 (1), 14–21. 10.70252/AYJX8444 27182398 PMC4831894

[B33] McHugh M. P. Cosgrave C. H. (2009). To stretch or not to stretch: the role of stretching in injury prevention and performance. Scand. J. Med. Sci. Sports 20, 169–181. 10.1111/j.1600-0838.2009.01058.x 20030776

[B34] Mika A. Mika P. Fernhall B. Unnithan V. B. (2007). Comparison of recovery strategies on muscle performance after fatiguing exercise. Am. J. Phys. Med. Rehabil. 86 (6), 474–481. 10.1097/PHM.0b013e31805b7c79 17515687

[B35] Mizumura K. Taguchi T. (2024). Neurochemical mechanism of muscular pain: insight from the study on delayed onset muscle soreness. J. Physiol. Sci. 74 (1), 4. 10.1186/s12576-023-00896-y 38267849 PMC10809664

[B36] Muanjai P. Namsawang J. (2015). Effects of stretching and cold-water immersion on functional signs of muscle soreness following plyometric training. J. Phys. Educ. Sport 15 (1), 128–135. 10.7752/jpes.2015.01021

[B37] Oba K. Samukawa M. Abe Y. Suzuki Y. Komatsuzaki M. Kasahara S. (2021). Effects of intermittent and continuous static stretching on range of motion and musculotendinous viscoelastic properties based on a duration-matched protocol. Int. J. Environ. Res. Public Health 18 (20), 10632. 10.3390/ijerph182010632 34682378 PMC8535970

[B38] Opplert J. Babault N. (2018). Acute effects of dynamic stretching on muscle flexibility and performance: an analysis of the current literature. Sports Med. 48 (2), 299–325. 10.1007/s40279-017-0797-9 29063454

[B39] Ozmen T. Gunes G. Y. Dogan H. Ucar I. Willems M. (2017). The effect of kinesio taping versus stretching techniques on muscle soreness, and flexibility during recovery from nordic hamstring exercise. J. Bodyw. Mov. Ther. 21 (1), 41–47. 10.1016/j.jbmt.2016.04.001 28167188

[B40] Ozsoy S. Conduit R. Moffitt R. Buszard T. Nash M. (2024). Exploring the perceptions and utilization of virtual reality in tennis coaching: insights from high-performance Australian coaches. J. Clin. Exerc Physiol. 13 (S2), 303. 10.31189/2165-7629-13-s2.303

[B41] Page M. J. McKenzie J. E. Bossuyt P. M. Boutron I. Hoffmann T. C. Mulrow C. D. (2021). The PRISMA 2020 statement: an updated guideline for reporting systematic reviews. BMJ n71, n71. 10.1136/bmj.n71 33782057 PMC8005924

[B42] Pooley S. Spendiff O. Allen M. Moir H. J. (2017). Static stretching does not enhance recovery in elite youth soccer players. BMJ Open Sport Exerc Med. 3 (1), e000202. 10.1136/bmjsem-2016-000202 28761702 PMC5530097

[B43] Pooley S. Spendiff O. Allen M. Moir H. J. (2020). Comparative efficacy of active recovery and cold water immersion as post-match recovery interventions in elite youth soccer. J. Sports Sci. 38 (11–12), 1423–1431. 10.1080/02640414.2019.1660448 31456474

[B44] Prabhu R. K. Swaminathan N. Harvey L. A. (2013). Passive movements for the treatment and prevention of contractures. Cochrane Database Syst. Rev. 2013 (1), CD009331. Available online at: http://doi.wiley.com/10.1002/14651858.CD009331.pub2. 24374605 10.1002/14651858.CD009331.pub2 PMC11001291

[B45] Sedano S. Maroto-Izquierdo S. (2025). Effectiveness of different neuromuscular recovery strategies in elite youth female football players. Sports 13 (2), 36. 10.3390/sports13020036 39997967 PMC11860187

[B46] Sohail M. A. A. Tahir R. Maqbool A. Hanif S. Saeed O. (2022). Comparing the effectiveness of static stretching and proprioceptive neuromuscular facilitation stretching in treating delayed onset muscle soreness in calf muscles of runners. Anaesth. Pain Intensive Care 26 (1), 31–38. 10.35975/apic.v26i1.1763

[B47] Støve M. P. Hirata R. P. Palsson T. S. (2024a). Regional and widespread pain sensitivity decreases following stretching in both men and women – indications of stretch-induced hypoalgesia. J. Bodyw. Mov. Ther. 39, 32–37. 10.1016/j.jbmt.2024.02.003 38876646

[B48] Støve M. P. Thomsen J. L. Magnusson S. P. Riis A. (2024b). The effect of six-week regular stretching exercises on regional and distant pain sensitivity: an experimental longitudinal study on healthy adults. BMC Sports Sci. Med. Rehabil. 16 (1), 202. 10.1186/s13102-024-00995-2 39334218 PMC11437648

[B49] Støve M. P. Hansen L. Ø. Elmbæk K. K. Magnusson S. P. Thomsen J. L. Riis A. (2025). The effect of stretching intensity on pain sensitivity: a randomized crossover study on healthy adults. Eur. J. Pain 29 (3), e4750. 10.1002/ejp.4750 39460597 PMC11755707

[B50] Takeuchi K. Nakamura M. Kakihana H. Tsukuda F. (2019). A survey of static and dynamic stretching protocol. Int. J. Sport Health Sci. 17 (0), 72–79. 10.5432/ijshs.201829

[B51] Takeuchi K. Nakamura M. Fukaya T. Konrad A. Mizuno T. (2023). Acute and long-term effects of static stretching on muscle-Tendon unit stiffness: a systematic review and meta-analysis. J. Sports Sci. Med. 22, 465–475. 10.52082/jssm.2023.465 37711702 PMC10499138

[B52] Thacker S. B. Gilchrist J. Stroup D. F. Kimsey C. D. (2004). The impact of stretching on sports injury risk: a systematic review of the literature. Med. Sci. Sports Exerc 36 (3), 371–378. 10.1249/01.mss.0000117134.83018.f7 15076777

[B53] Torres R. Pinho F. Duarte J. A. Cabri J. M. H. (2013). Effect of single bout versus repeated bouts of stretching on muscle recovery following eccentric exercise. J. Sci. Med. Sport 16 (6), 583–588. 10.1016/j.jsams.2013.01.002 24139151

[B54] Trajano G. S. Nosaka K. Blazevich A. J. (2017). Neurophysiological mechanisms underpinning stretch-induced force loss. Sports Med. 47 (8), 1531–1541. 10.1007/s40279-017-0682-6 28120238

[B55] Vesterinen H. M. Sena E. S. Egan K. J. Hirst T. C. Churolov L. Currie G. L. (2014). Meta-analysis of data from animal studies: a practical guide. J. Neurosci. Methods 221, 92–102. 10.1016/j.jneumeth.2013.09.010 24099992

[B56] Warneke K. Brinkmann A. Hillebrecht M. Schiemann S. (2022). Influence of long-lasting static stretching on maximal strength, muscle thickness and flexibility. Front. Physiol. 13, 878955. 10.3389/fphys.2022.878955 35694390 PMC9174468

[B57] Warneke K. Lohmann L. H. Plöschberger G. Konrad A. (2024a). Critical evaluation and recalculation of current systematic reviews with meta-analysis on the effects of acute and chronic stretching on passive properties and passive peak torque. Eur. J. Appl. Physiol. 124 (11), 3153–3173. 10.1007/s00421-024-05564-6 39066912 PMC11519181

[B58] Warneke K. Lohmann L. H. Behm D. G. Wirth K. Keiner M. Schiemann S. (2024b). Effects of chronic static stretching on maximal strength and muscle hypertrophy: a systematic review and meta-analysis with meta-regression. Sports Med. - Open 10 (1), 45. 10.1186/s40798-024-00706-8 38637473 PMC11026323

[B59] Warneke K. Konrad A. Wilke J. (2024c). The knowledge of movement experts about stretching effects: does the science reach practice? Ramskov D. PLOS ONE 19 (1), e0295571. 10.1371/journal.pone.0295571 38277378 PMC10817148

[B60] Wessel J. Wan A. (1994). Effect of stretching on the intensity of delayed-onset muscle soreness. Clin. J. Sport Med. 4 (2), 83–87. 10.1097/00042752-199404000-00003

[B61] West A. D. Cooke M. B. LaBounty P. M. Byars A. G. Greenwood M. (2014). Effects of G-Trainer, cycle ergometry, and stretching on physiological and psychological recovery from endurance exercise. J. Strength Cond. Res. 28 (12), 3453–3461. 10.1519/JSC.0000000000000577 24936899

[B62] Xie Y. Feng B. Chen K. Andersen L. L. Page P. Wang Y. (2018). The efficacy of dynamic contract-relax stretching on delayed-onset muscle soreness among healthy individuals: a randomized clinical trial. Clin. J. Sport Med. 28 (1), 28–36. 10.1097/JSM.0000000000000442 28742609

[B63] Yan J. Wang W. B. Fan Y. J. Bao H. Li N. Yao Q. P. (2020). Cyclic stretch induces vascular smooth muscle cells to secrete connective tissue growth factor and promote endothelial progenitor cell differentiation and angiogenesis. Front. Cell Dev. Biol. 8, 606989. 10.3389/fcell.2020.606989 33363166 PMC7755638

[B64] Zedka M. Prochazka A. Knight B. Gillard D. Gauthier M. (1999). Voluntary and reflex control of human back muscles during induced pain. J. Physiol. 520 (2), 591–604. 10.1111/j.1469-7793.1999.00591.x 10523425 PMC2269584

[B65] Zvetkova E. Koytchev E. Ivanov I. Ranchev S. Antonov A. (2023). Biomechanical, healing and therapeutic effects of stretching: a comprehensive review. Appl. Sci. 13 (15), 8596. 10.3390/app13158596

