# Supplementary material for: Effects of post-exercise stretching versus no stretching on lower limb muscle recovery and performance: a meta-analysis
Source: Front Physiol. 2025 Oct 1;16:1674871. doi: 10.3389/fphys.2025.1674871 (PMC12521117; doi:10.3389/fphys.2025.1674871)
Supplement: Supplementary file 1 [file Supplementaryfile6.docx]

**Date:** 2025-08-28
**Question:** Effects of post-Exercise Stretching versus No Stretching on Lower Limb Muscle Recovery and performance: A Meta-Analysis
**Bibliography:**

| **Quality assessment** | | | | | | | **Quality** | **Importance** |
| --- | --- | --- | --- | --- | --- | --- | --- | --- |
|  |  |  |  |  |  |  |  |  |
| **No of studies** | **Design** | **Risk of bias** | **Inconsistency** | **Indirectness** | **Imprecision** | **Other considerations** |  |  |
| 17 | randomised trials | serious^1^ | no serious inconsistency | no serious indirectness | serious^1^ | none | ⊕⊕OO LOW | CRITICAL |
|  |  |  |  |  |  |  |  |  |
| 6 | randomised trials | serious^1^ | no serious inconsistency | no serious indirectness | serious^1^ | none | ⊕⊕OO LOW | CRITICAL |
|  |  |  |  |  |  |  |  |  |
| 5 | randomised trials | serious^1^ | no serious inconsistency^1^ | no serious indirectness | serious^1^ | none | ⊕⊕OO LOW |  |
|  |  |  |  |  |  |  |  |  |
| 11 | randomised trials | serious^1^ | no serious inconsistency | no serious indirectness | serious^1^ | none | ⊕⊕OO LOW |  |
|  |  |  |  |  |  |  |  |  |
| 6 | randomised trials | serious^1^ | no serious inconsistency | no serious indirectness | serious^1^ | none | ⊕⊕OO LOW |  |
|  |  |  |  |  |  |  |  |  |

^1^ No explanation was provided
