# Supplementary material for: Effects of post-exercise stretching versus no stretching on lower limb muscle recovery and performance: a meta-analysis
Source: Front Physiol. 2025 Oct 1;16:1674871. doi: 10.3389/fphys.2025.1674871 (PMC12521117; doi:10.3389/fphys.2025.1674871)
Supplement: Supplementary file 2 [file Supplementaryfile5.docx]

**Leave-one-out sensitivity analyses for all outcomes**

**Leave-one-out sensitivity analyses**

**
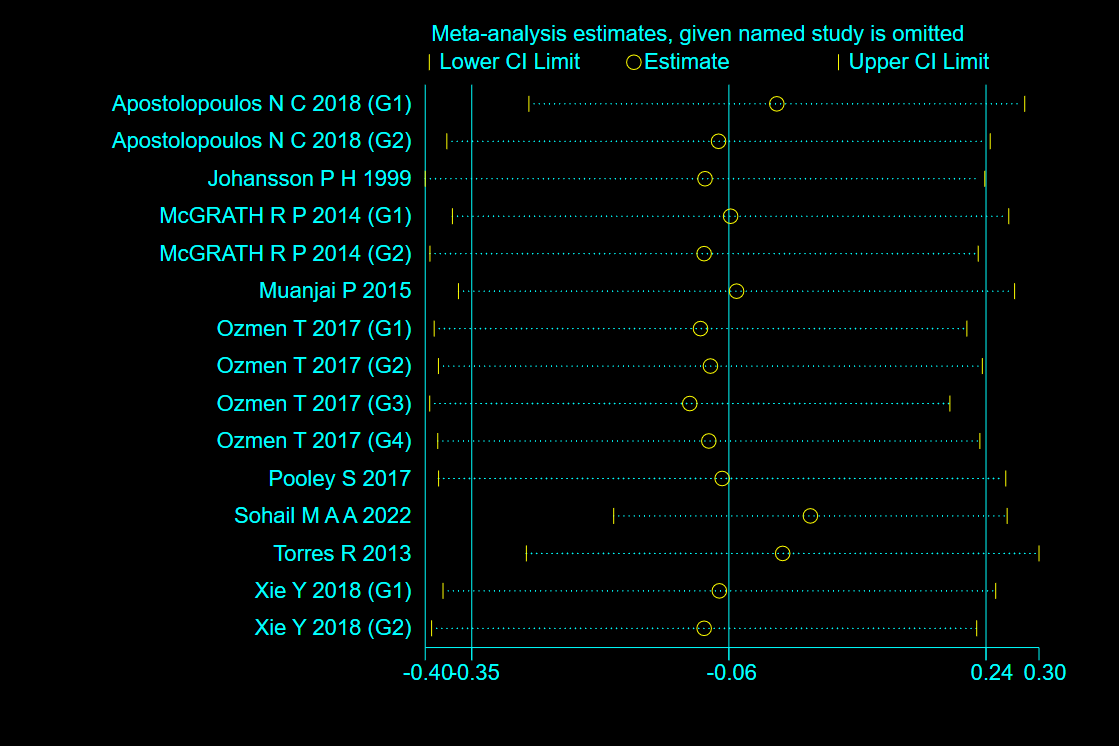
**

**Figure 1.** Leave-one-out sensitivity analysis for muscle soreness.

**
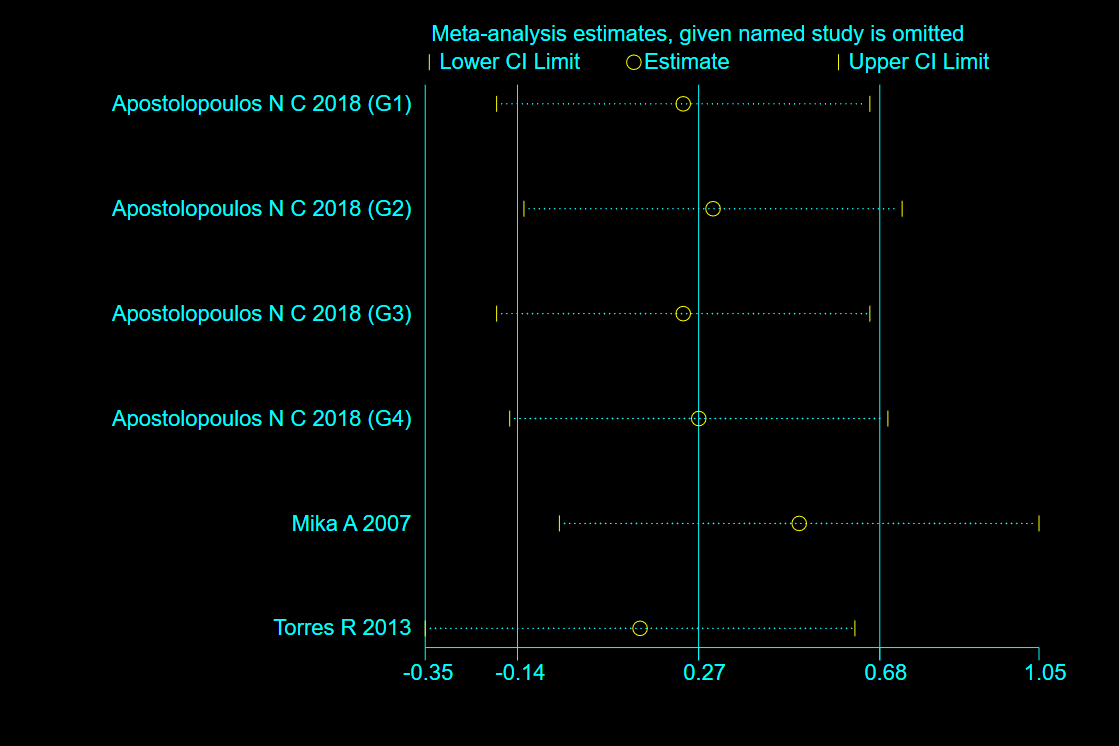
**Figure 2. Leave-one-out sensitivity analysis for strength.**
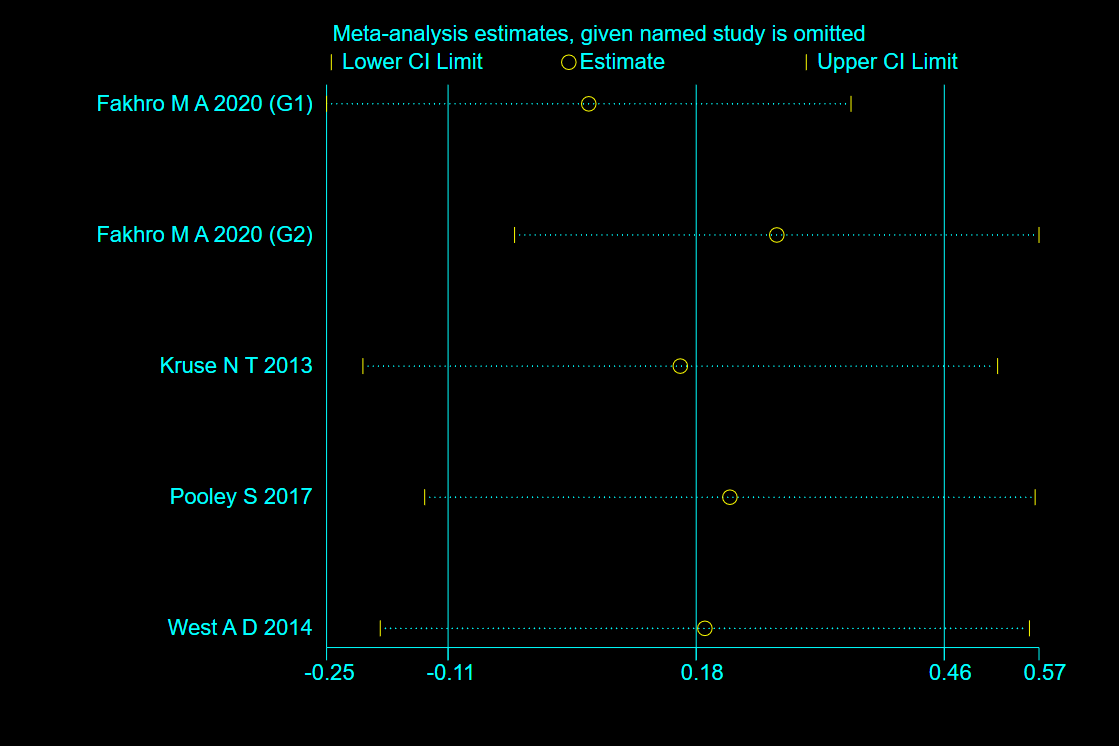
**

Figure 3. Leave-one-out sensitivity analysis for performance.

**
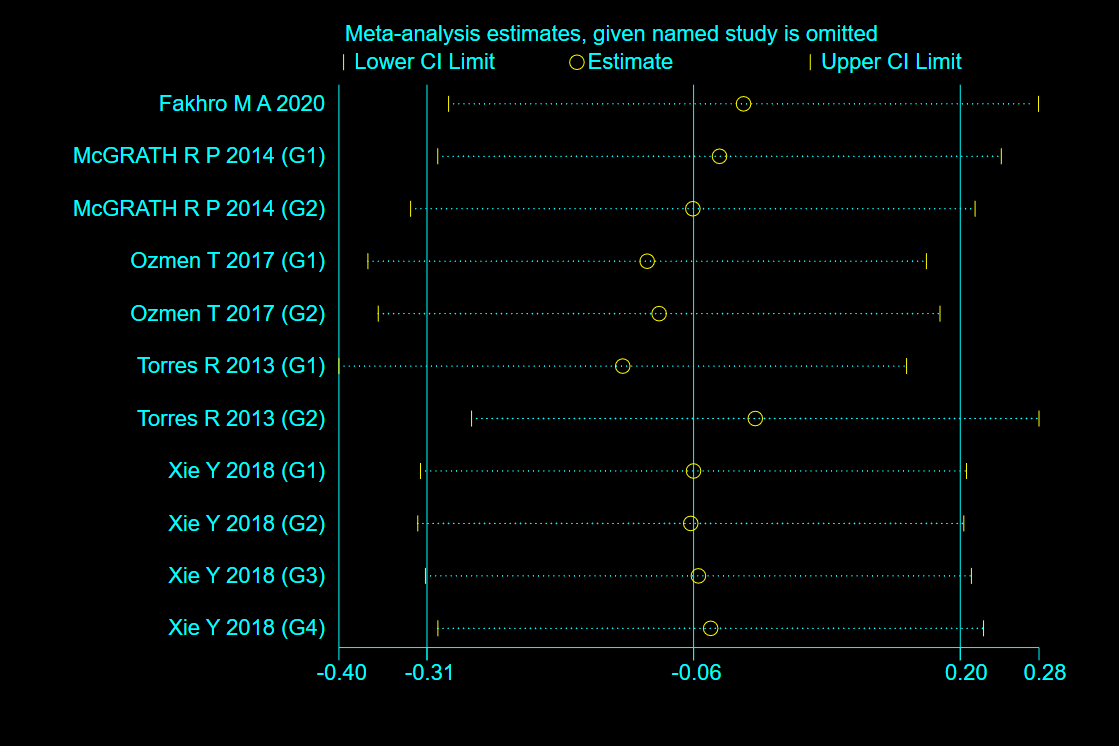
**

Figure 4. Leave-one-out sensitivity analysis for flexibility.

**
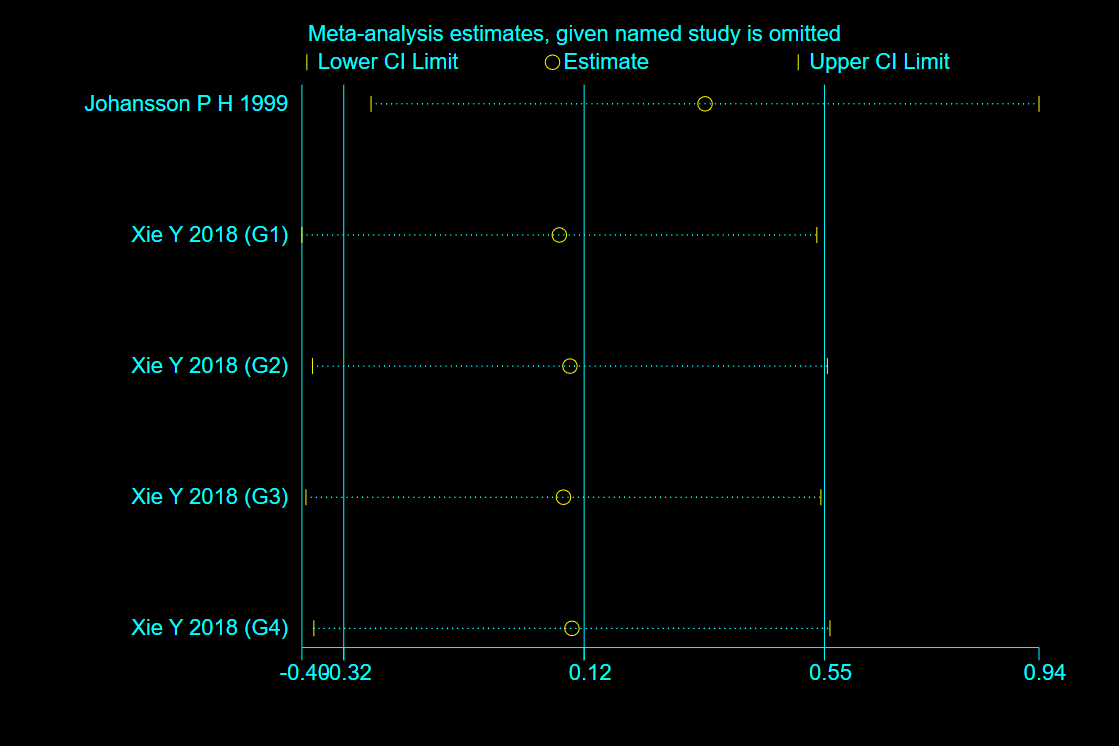
**

Figure 5. Leave-one-out sensitivity analysis for pain threshold.

**Meta-regression analyses**

**
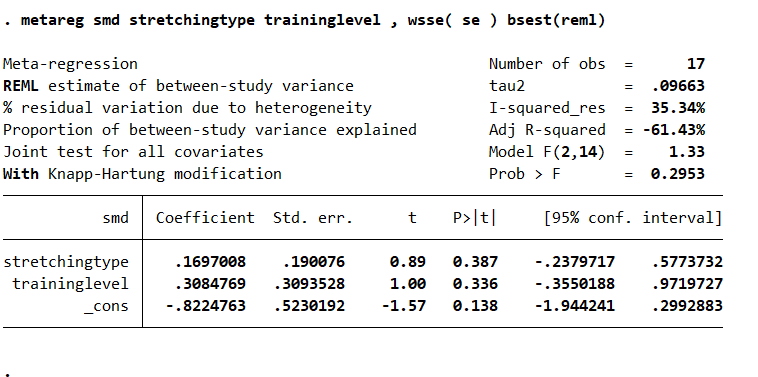
**

Figure 6. Meta-regression for muscle soreness

**
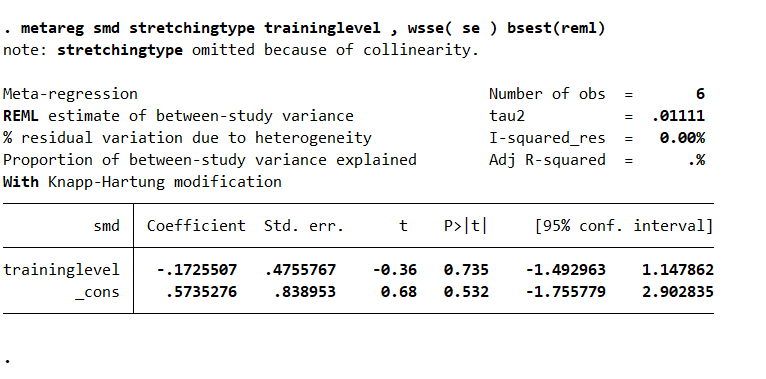
**

Figure 7. Meta-regression for strength.

**
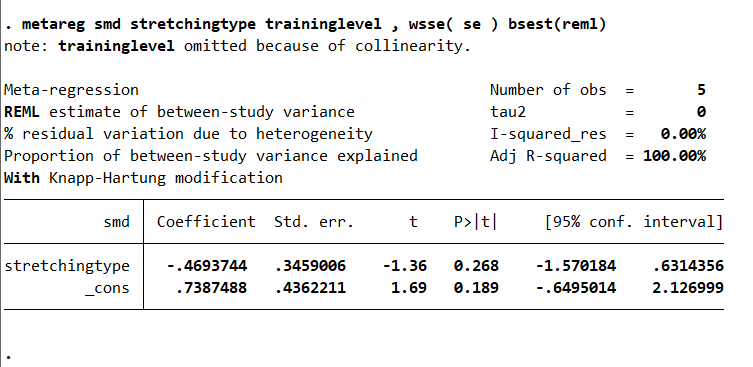
**

Figure 8. Meta-regression for performance.

**
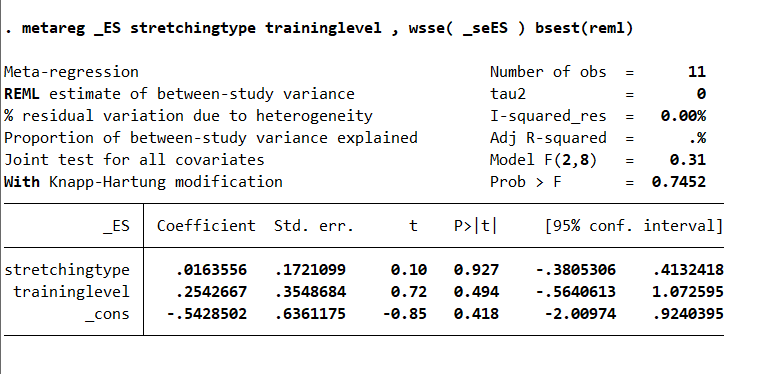
**

Figure 9. Meta-regression for flexibility.

**
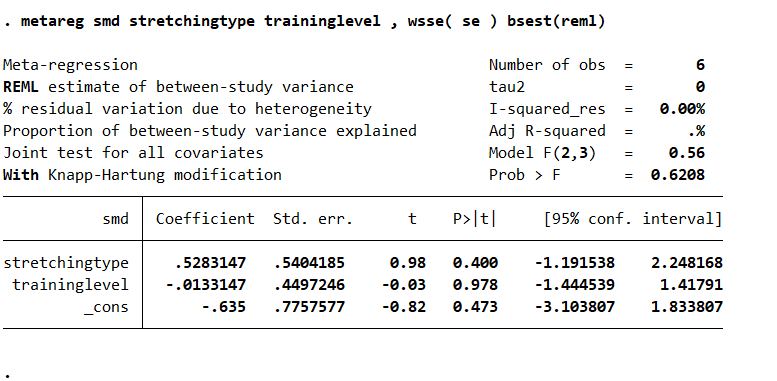
**

Figure 10. Meta-regression for pain threshold.
