# Supplementary material for: Effects of post-exercise stretching versus no stretching on lower limb muscle recovery and performance: a meta-analysis
Source: Front Physiol. 2025 Oct 1;16:1674871. doi: 10.3389/fphys.2025.1674871 (PMC12521117; doi:10.3389/fphys.2025.1674871)
Supplement: Supplementary file 4 [file Supplementaryfile3.docx]

**Appendix B Cochrane risk-of-bias tool for randomized trials (RoB 2)**

**Author(s):**
**Date:** 2025-07-25

Table 1. Randomized Controlled Trial Score Sheet.

| Author | Year | Bias arising from the randomization process | | Bias due to deviations from intended interventions | | Bias due to missing outcome data | | Bias in measurement of the outcome | | Bias in selection of the reported result | | Overall risk of bias |
| --- | --- | --- | --- | --- | --- | --- | --- | --- | --- | --- | --- | --- |
|  |  | risk-of-bias judgement | Reasons for judgement | risk-of-bias judgement | Reasons for judgement | risk-of-bias judgement | Reasons for judgement | risk-of-bias judgement | Reasons for judgement | risk-of-bias judgement | Reasons for judgement |  |
| Apostolopoulos N C | 2018 | Low | Adequate random sequence generation and concealment described. | Some concerns | Blind methods are not implemented and intervention deviations may occur. | Low | Data for this outcome were available for all, or nearly all, participants randomized or less than 5% of data were missing with balanced dropout. | Some concerns | The result measurement part was self-assessment by the subjects, which was subject to subjective bias. | Low | All outcomes listed in the protocol were reported. | Some concerns |
| Sohail M A A | 2022 | Low | Adequate random sequence generation and concealment described. | Some concerns | Blind methods are not implemented and intervention deviations may occur. | Low | Data for this outcome were available for all, or nearly all, participants randomized or less than 5% of data were missing with balanced dropout. | Some concerns | The result measurement part was self-assessment by the subjects, which was subject to subjective bias. | Low | All outcomes listed in the protocol were reported. | Some concerns |
| Fakhro M A | 2020 | Low | Adequate random sequence generation and concealment described. | Low | No significant intervention deviations occurred during the course of the experiment; study participants were blinded and staff were not blinded but followed the designated intervention protocol; and the method of analysis was intentional. | Low | Data for this outcome were available for all, or nearly all, participants randomized or less than 5% of data were missing with balanced dropout. | Low | Measures of outcomes between intervention groups were appropriate, consistent and objective, with little assessor influence. | Low | All outcomes listed in the protocol were reported. | Low |
| Torres R | 2013 | Low | Adequate random sequence generation and concealment described. | Low | No significant intervention deviations occurred during the course of the experiment; study participants were blinded and staff were not blinded but followed the designated intervention protocol; and the method of analysis was intentional. | Low | Data for this outcome were available for all, or nearly all, participants randomized or less than 5% of data were missing with balanced dropout. | Low | Measures of outcomes between intervention groups were appropriate, consistent and objective, with little assessor influence. | Low | All outcomes listed in the protocol were reported. | Low |
| Ozmen T | 2017 | Some concerns | Randomization method was not described, leading to some concerns about allocation concealment. | Low | No significant intervention deviations occurred during the course of the experiment; study participants were blinded and staff were not blinded but followed the designated intervention protocol; and the method of analysis was intentional. | Low | Data for this outcome were available for all, or nearly all, participants randomized or less than 5% of data were missing with balanced dropout. | Some concerns | The result measurement part was self-assessment by the subjects, which was subject to subjective bias. | Low | All outcomes listed in the protocol were reported. | Some concerns |
| Xie Y | 2018 | Low | Adequate random sequence generation and concealment described. | Some concerns | Blind methods are not implemented and intervention deviations may occur. | Low | Data for this outcome were available for all, or nearly all, participants randomized or less than 5% of data were missing with balanced dropout. | Some concerns | The result measurement part was self-assessment by the subjects, which was subject to subjective bias. | Low | All outcomes listed in the protocol were reported. | Some concerns |
| Muanjai P | 2015 | Some concerns | Randomization method was not described, leading to some concerns about allocation concealment. | Low | No significant intervention deviations occurred during the course of the experiment; study participants were blinded and staff were not blinded but followed the designated intervention protocol; and the method of analysis was intentional. | Low | Data for this outcome were available for all, or nearly all, participants randomized or less than 5% of data were missing with balanced dropout. | Some concerns | The result measurement part was self-assessment by the subjects, which was subject to subjective bias. | Low | All outcomes listed in the protocol were reported. | Some concerns |
| McGRATH R P | 2014 | Some concerns | Randomization method was not described, leading to some concerns about allocation concealment. | Some concerns | Blind methods are not implemented and intervention deviations may occur. | Low | Data for this outcome were available for all, or nearly all, participants randomized or less than 5% of data were missing with balanced dropout. | Some concerns | The result measurement part was self-assessment by the subjects, which was subject to subjective bias. | Low | All outcomes listed in the protocol were reported. | Some concerns |
| McGlynn G H | 1979 | Some concerns | Randomization method was not described, leading to some concerns about allocation concealment. | Some concerns | Blind methods are not implemented and intervention deviations may occur. | Low | Data for this outcome were available for all, or nearly all, participants randomized or less than 5% of data were missing with balanced dropout. | High | No blinding was carried out, and the degree of subjectivity bias in the scores given by the participants was relatively high. | Low | All outcomes listed in the protocol were reported. | High |
| Wessel J | 1994 | Low | Adequate random sequence generation and concealment described. | Some concerns | Blind methods are not implemented and intervention deviations may occur. | Low | Data for this outcome were available for all, or nearly all, participants randomized or less than 5% of data were missing with balanced dropout. | High | No blinding was carried out, and the degree of subjectivity bias in the scores given by the participants was relatively high. | Low | All outcomes listed in the protocol were reported. | High |

Table 2. Randomized crossover trial scoring sheet.

| Author | Year | Bias arising from the randomization process | | Bias due to deviations from intended interventions | | Bias due to missing outcome data | | Bias in measurement of the outcome | | Bias in selection of the reported result | | Bias arising from period and carryover effects | | Overall risk of bias |
| --- | --- | --- | --- | --- | --- | --- | --- | --- | --- | --- | --- | --- | --- | --- |
|  |  | risk-of-bias judgement | Reasons for judgement | risk-of-bias judgement | Reasons for judgement | risk-of-bias judgement | Reasons for judgement | risk-of-bias judgement | Reasons for judgement | risk-of-bias judgement | Reasons for judgement | risk-of-bias judgement | Reasons for judgement |  |
| Lund H | 1998 | Some concerns | Randomization method was not described, leading to some concerns about allocation concealment. | Some concerns | Blind methods are not implemented and intervention deviations may occur. | Low | Data for this outcome were available for all, or nearly all, participants randomized or less than 5% of data were missing with balanced dropout. | Some concerns | The result measurement part was self-assessment by the subjects, which was subject to subjective bias. | Low | All outcomes listed in the protocol were reported. | High | There is a confounding effect. | High |
| Kruse N T | 2013 | Low | Adequate random sequence generation and concealment described. | Low | No significant intervention deviations occurred during the course of the experiment; study participants were blinded and staff were not blinded but followed the designated intervention protocol; and the method of analysis was intentional. | Low | Data for this outcome were available for all, or nearly all, participants randomized or less than 5% of data were missing with balanced dropout. | Low | Measures of outcomes between intervention groups were appropriate, consistent and objective, with little assessor influence. | Low | All outcomes listed in the protocol were reported. | Low | Eliminated confounding effects | Low |
| Pooley S | 2017 | Some concerns | Randomization method was not described, leading to some concerns about allocation concealment. | Low | No significant intervention deviations occurred during the course of the experiment; study participants were blinded and staff were not blinded but followed the designated intervention protocol; and the method of analysis was intentional. | Low | Data for this outcome were available for all, or nearly all, participants randomized or less than 5% of data were missing with balanced dropout. | Some concerns | The result measurement part was self-assessment by the subjects, which was subject to subjective bias. | Low | All outcomes listed in the protocol were reported. | Some concerns | There may be confounding effects that lead to bias. | Some concerns |
| West A D | 2014 | Low | Adequate random sequence generation and concealment described. | Low | No significant intervention deviations occurred during the course of the experiment; study participants were blinded and staff were not blinded but followed the designated intervention protocol; and the method of analysis was intentional. | Low | Data for this outcome were available for all, or nearly all, participants randomized or less than 5% of data were missing with balanced dropout. | Some concerns | The result measurement part was self-assessment by the subjects, which was subject to subjective bias. | Low | All outcomes listed in the protocol were reported. | Low | Eliminated confounding effects | Some concerns |
| Mika A | 2007 | Low | Adequate random sequence generation and concealment described. | Low | No significant intervention deviations occurred during the course of the experiment; study participants were blinded and staff were not blinded but followed the designated intervention protocol; and the method of analysis was intentional. | Low | Data for this outcome were available for all, or nearly all, participants randomized or less than 5% of data were missing with balanced dropout. | Low | Measures of outcomes between intervention groups were appropriate, consistent and objective, with little assessor influence. | Low | All outcomes listed in the protocol were reported. | Some concerns | There may be confounding effects that lead to bias. | Some concerns |
| Johansson P H | 1999 | Low | Adequate random sequence generation and concealment described. | Low | No significant intervention deviations occurred during the course of the experiment; study participants were blinded and staff were not blinded but followed the designated intervention protocol; and the method of analysis was intentional. | Low | Data for this outcome were available for all, or nearly all, participants randomized or less than 5% of data were missing with balanced dropout. | Low | Measures of outcomes between intervention groups were appropriate, consistent and objective, with little assessor influence. | Low | All outcomes listed in the protocol were reported. | Some concerns | There may be confounding effects that lead to bias. | Some concerns |
