# Supplementary material for: Effects of post-exercise stretching versus no stretching on lower limb muscle recovery and performance: a meta-analysis
Source: Front Physiol. 2025 Oct 1;16:1674871. doi: 10.3389/fphys.2025.1674871 (PMC12521117; doi:10.3389/fphys.2025.1674871)
Supplement: Supplementary file 5 [file Supplementaryfile2.docx]

**Appendix B Database Search Terms**

##

## Literature Search Strategy for Web of Science

| **Search Component** | **Search Terms** |
| --- | --- |
| **Population** | TS=("static stretch*" OR "passive stretch*" OR "post-exercise stretch*") |
| **Intervention (IHT-related terms)** | TS=("muscle recover*" OR DOMS OR "muscle strength" OR "vertical jump") |
| **Study Type** | TS=("lower limb*" OR quadriceps OR hamstring* OR calf OR performance) |

## Literature Search Strategy for Scoup

| **Search Component** | **Search Terms** |
| --- | --- |
| **Population** | TITLE-ABS-KEY ( "static stretch*" OR "passive stretch*" OR "post-exercise stretch*" ) |
| **Intervention (IHT-related terms)** | TITLE-ABS-KEY ( "muscle recover*" OR DOMS OR "muscle strength" OR "vertical jump" ) |
| **Study Type** | TITLE-ABS-KEY ( "lower limb*" OR quadriceps OR hamstring* OR calf OR performance ) |

## Literature Search Strategy for PubMed

| **Search Component** | **Search Terms** |
| --- | --- |
| **Population** | ("static stretch*"[All Fields] OR "passive stretch*"[All Fields] OR "post-exercise stretch*"[All Fields]) |
| **Intervention (IHT-related terms)** | ("muscle recover*"[All Fields] OR "DOMS"[All Fields] OR "muscle strength"[All Fields] OR "vertical jump"[All Fields]) |
| **Study Type** | ("lower limb*"[All Fields] OR "quadriceps"[All Fields] OR "hamstring*"[All Fields] OR "calf"[All Fields] OR "performance"[All Fields]) |

## Literature Search Strategy for Embase:

| **Search Component** | **Search Terms** |
| --- | --- |
| **Population** | TITLE-ABS-KEY ( "static stretch*" OR "passive stretch*" OR "post-exercise stretch*" ) |
| **Intervention (IHT-related terms)** | TITLE-ABS-KEY ( "muscle recover*" OR DOMS OR "muscle strength" OR "vertical jump" ) |
| **Study Type** | TITLE-ABS-KEY ( "lower limb*" OR quadriceps OR hamstring* OR calf OR performance ) |

## Literature Search Strategy for Cochranelibrary:

| **Search Component** | **Search Terms** |
| --- | --- |
| **Population** | static stretch* OR passive stretch* OR post-exercise stretch* |
| **Intervention (IHT-related terms)** | muscle recover* OR DOMS OR muscle strength OR vertical jump |
| **Study Type** | lower limb* OR quadriceps OR hamstring* OR calf OR performance |

## Literature Search Strategy for SPORTDiscus:

| **Search Component** | **Search Terms** |
| --- | --- |
| **Population** | static stretch* OR passive stretch* OR post-exercise stretch* |
| **Intervention (IHT-related terms)** | muscle recover* OR DOMS OR muscle strength OR vertical jump |
| **Study Type** | lower limb* OR quadriceps OR hamstring* OR calf OR performance |
