# Supplementary material for: Effects of post-exercise stretching versus no stretching on lower limb muscle recovery and performance: a meta-analysis
Source: Front Physiol. 2025 Oct 1;16:1674871. doi: 10.3389/fphys.2025.1674871 (PMC12521117; doi:10.3389/fphys.2025.1674871)
Supplement: Supplementary file 6 [file Supplementaryfile4.docx]

**Appendix C Correlation Chart**


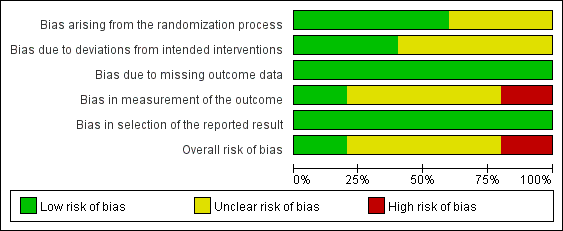


Figure 1 Proportion of randomised controlled trial rated as low risk, some concerns, or high risk in each RoB 2 domain.


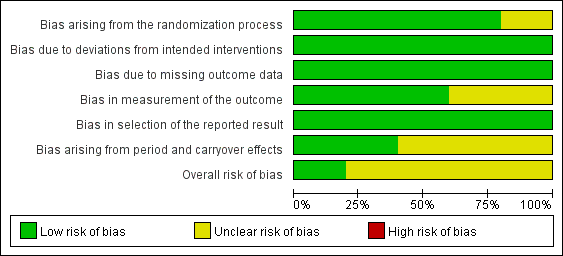


Figure 2 Proportion of randomised crossover trial rated as low risk, some concerns, or high risk in each RoB 2 domain.


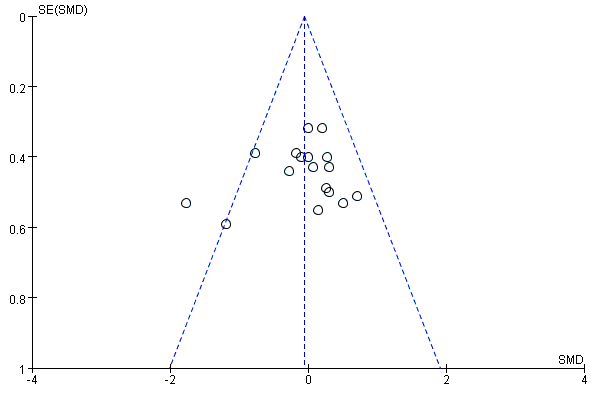


Figure 3 Funnel plot assessing publication bias in muscle soreness.


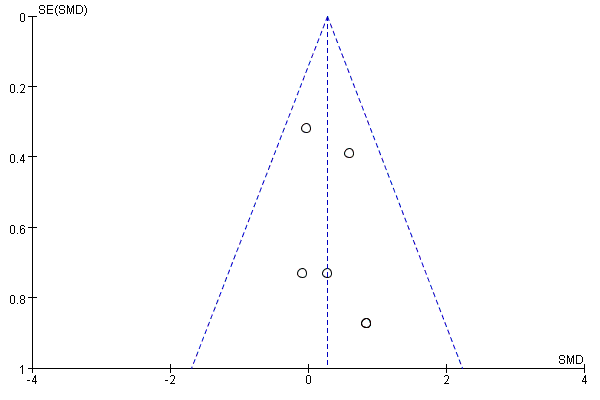


Figure 4 Funnel plot assessing publication bias in strength.


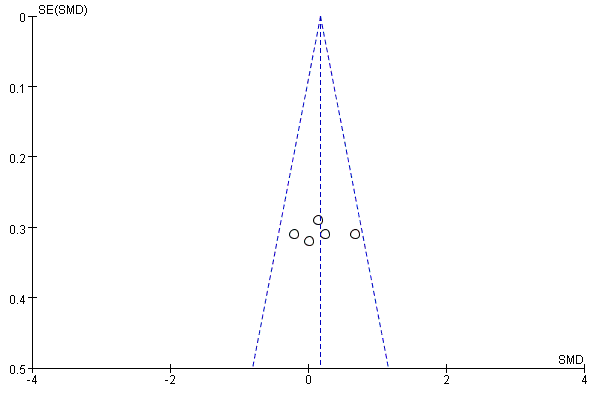


Figure 5 Funnel plot assessing publication bias in performance.


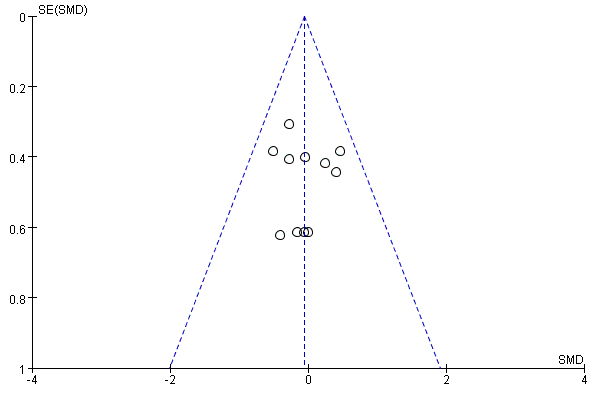


Figure 6 Funnel plot assessing publication bias in flexibility.


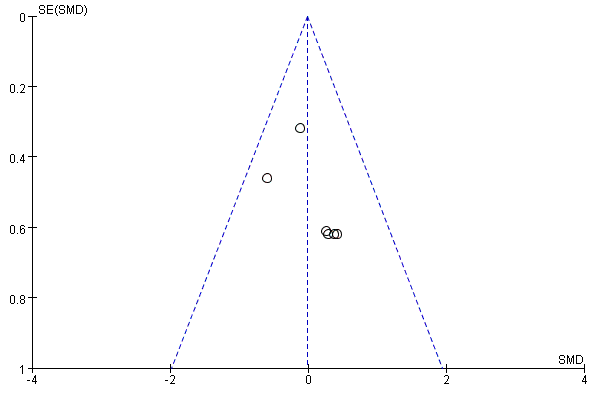


Figure 7 Funnel plot assessing publication bias in pain threshold.


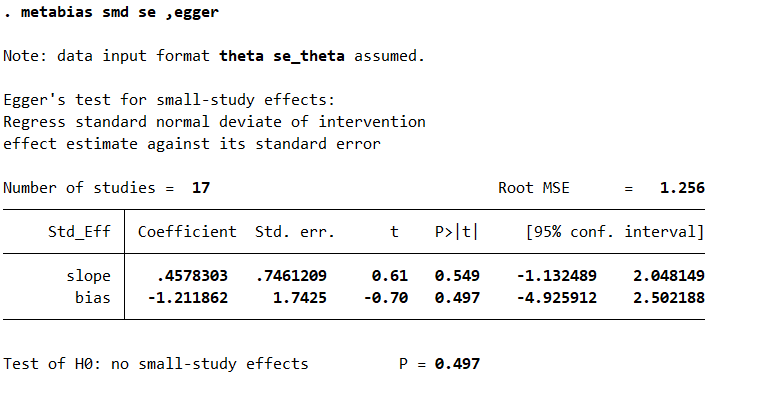


Figure 8 Egger’ test assessing publication bias in muscle soreness.


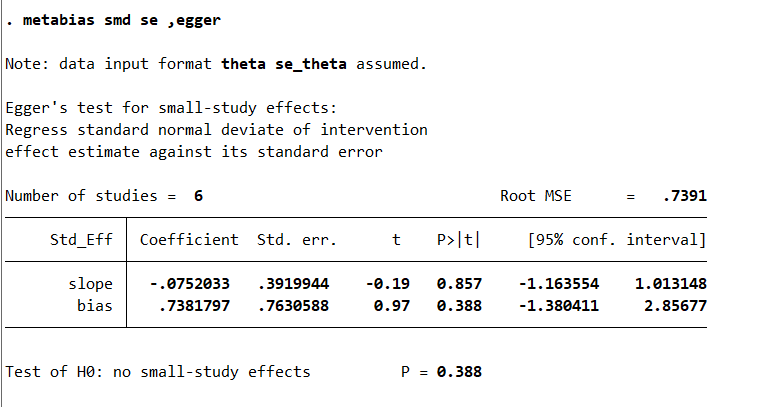


Figure 9 Egger’ test assessing publication bias in strength.


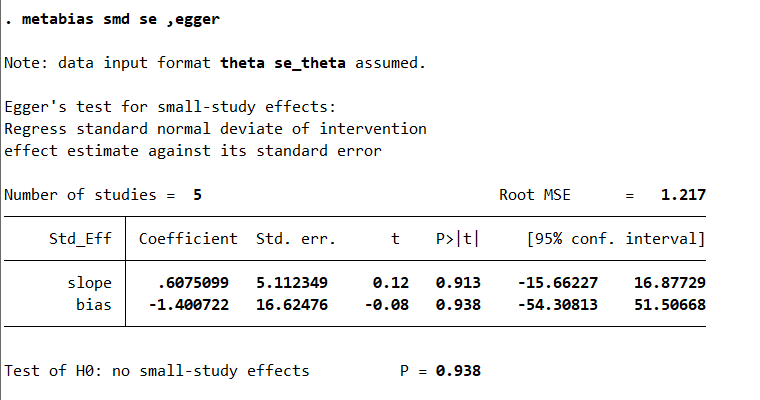


Figure 10 Egger’ test assessing publication bias in performance.


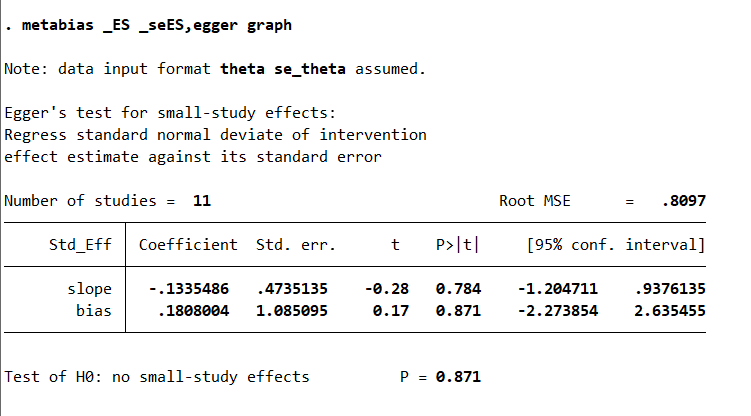


Figure 11 Egger’ test assessing publication bias in flexibility.


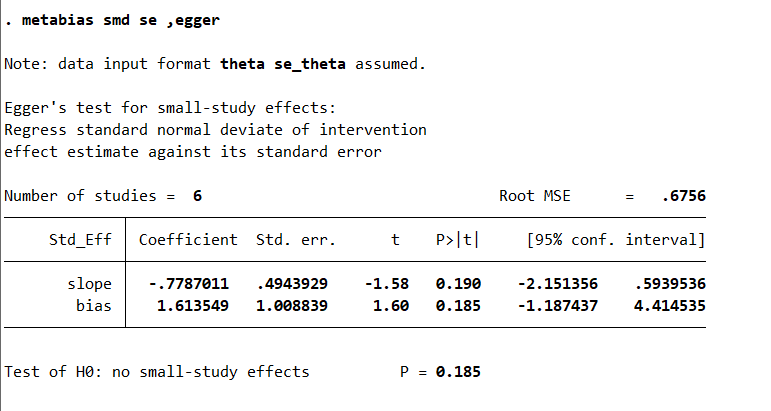


Figure 12 Egger’ test assessing publication bias in pain threshold.


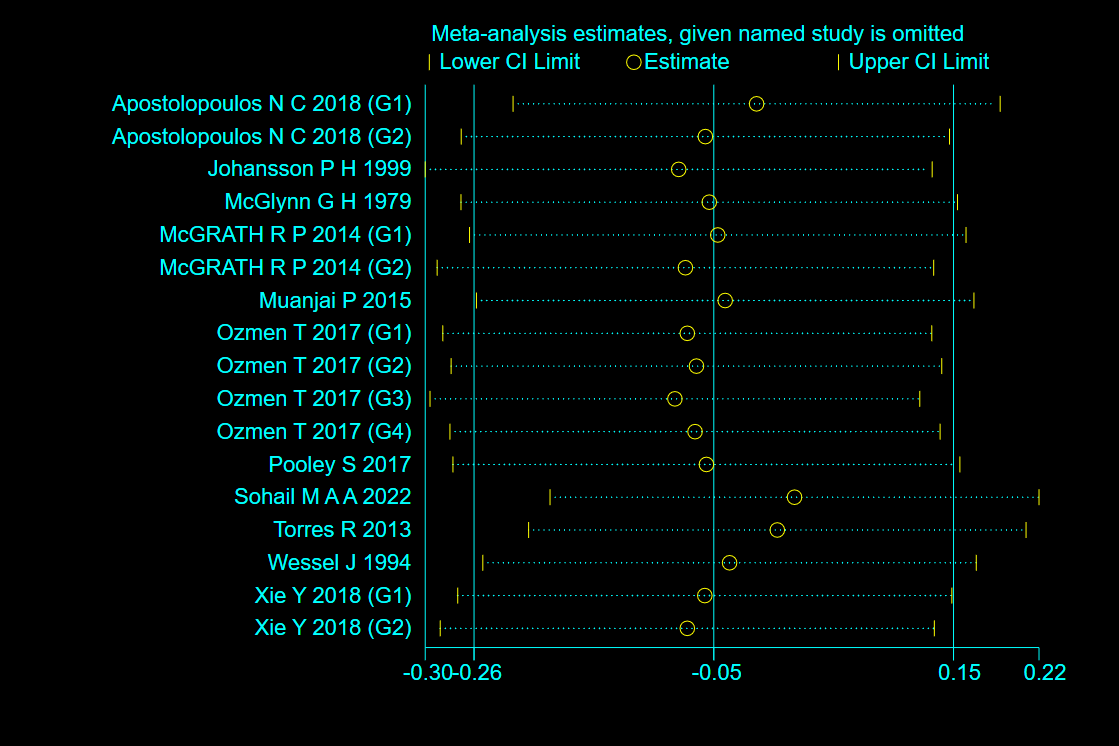


Figure 13 Leave-one-out sensitivity analysis in muscle soreness.


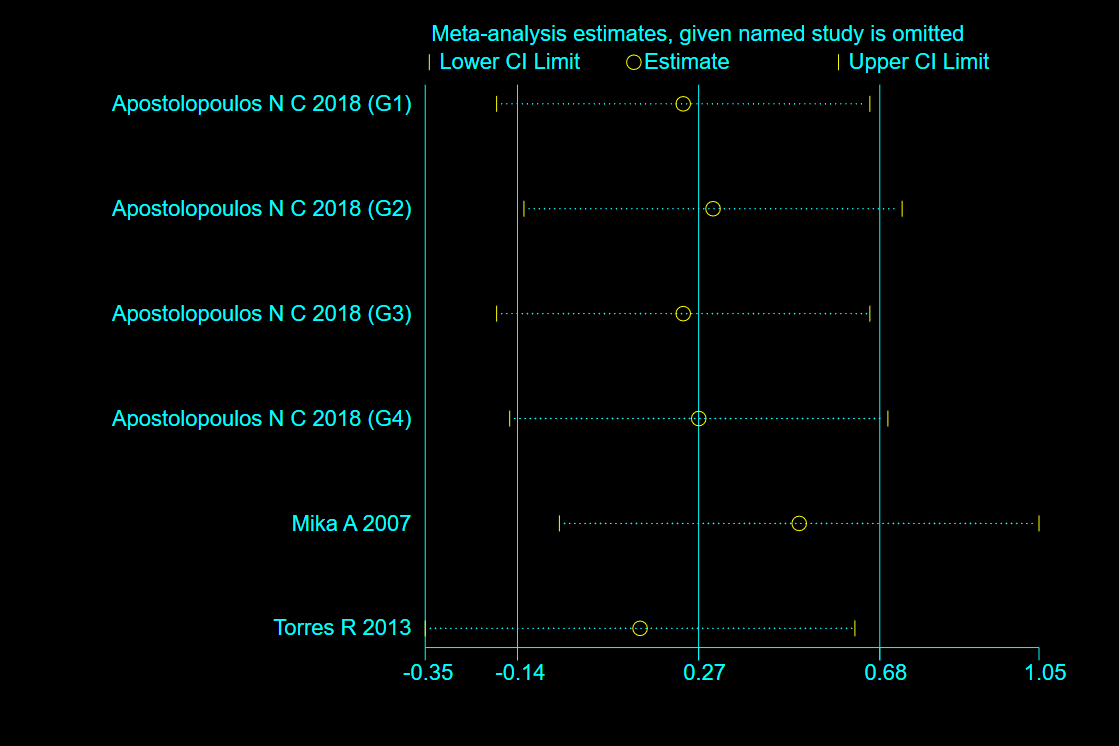


Figure 14 Leave-one-out sensitivity analysis in strength.


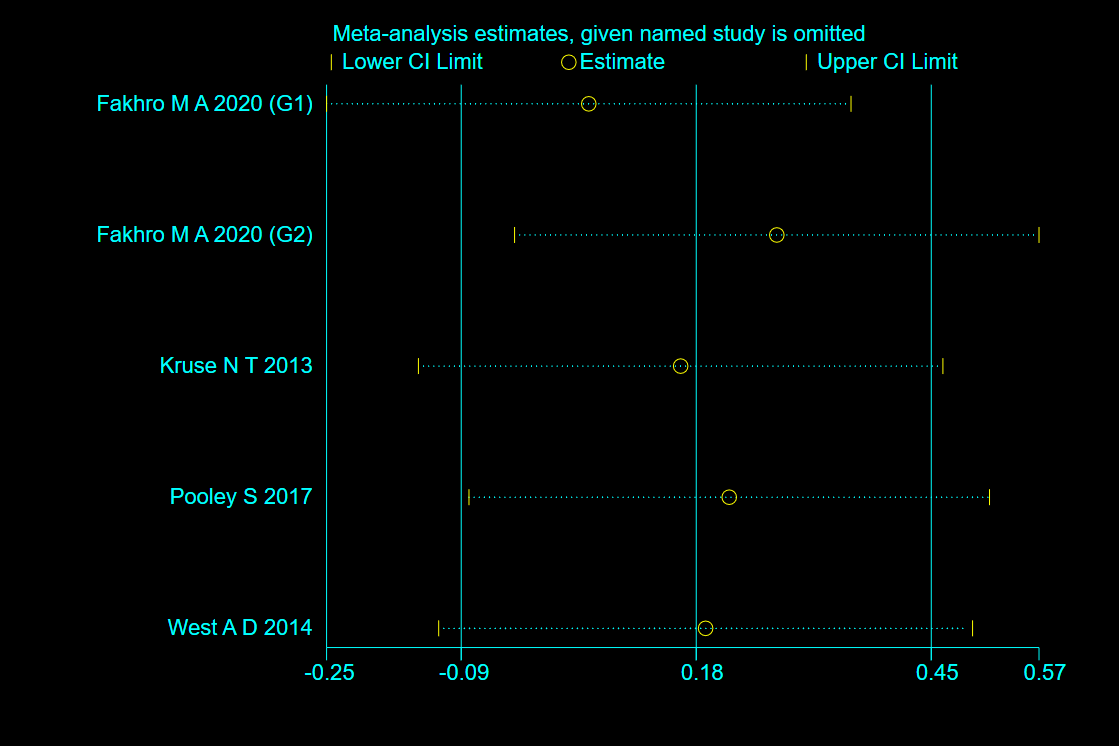


Figure 15 Leave-one-out sensitivity analysis in performance.


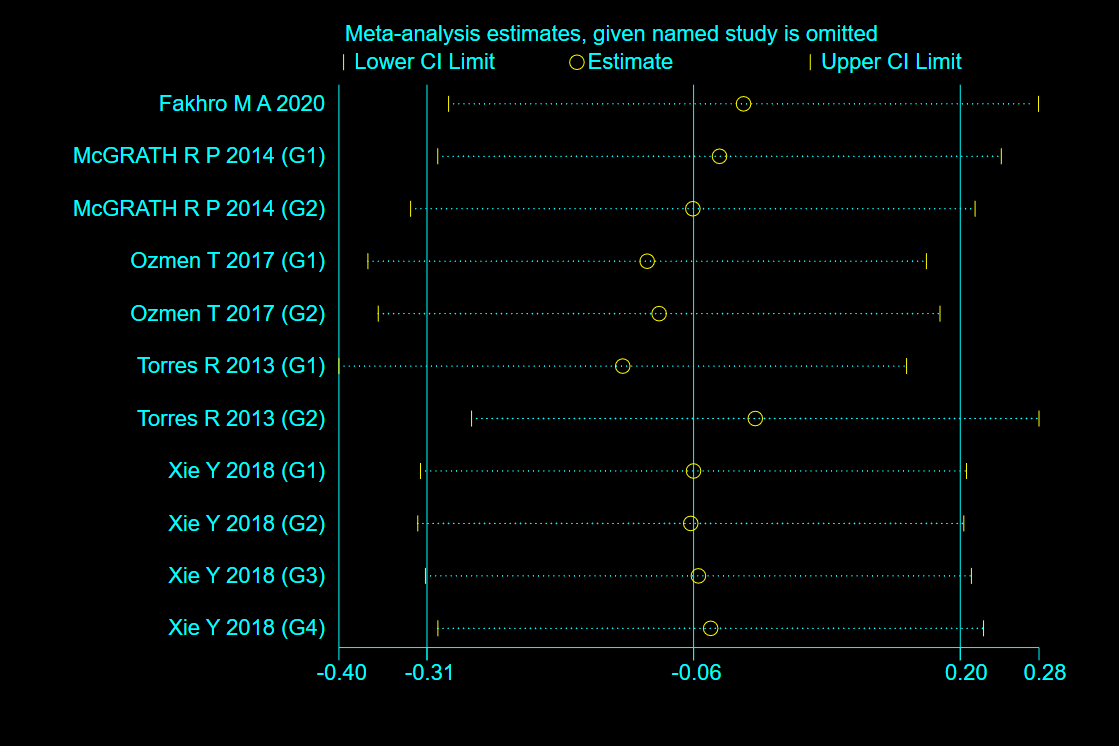


Figure 16 Leave-one-out sensitivity analysis in flexibility.


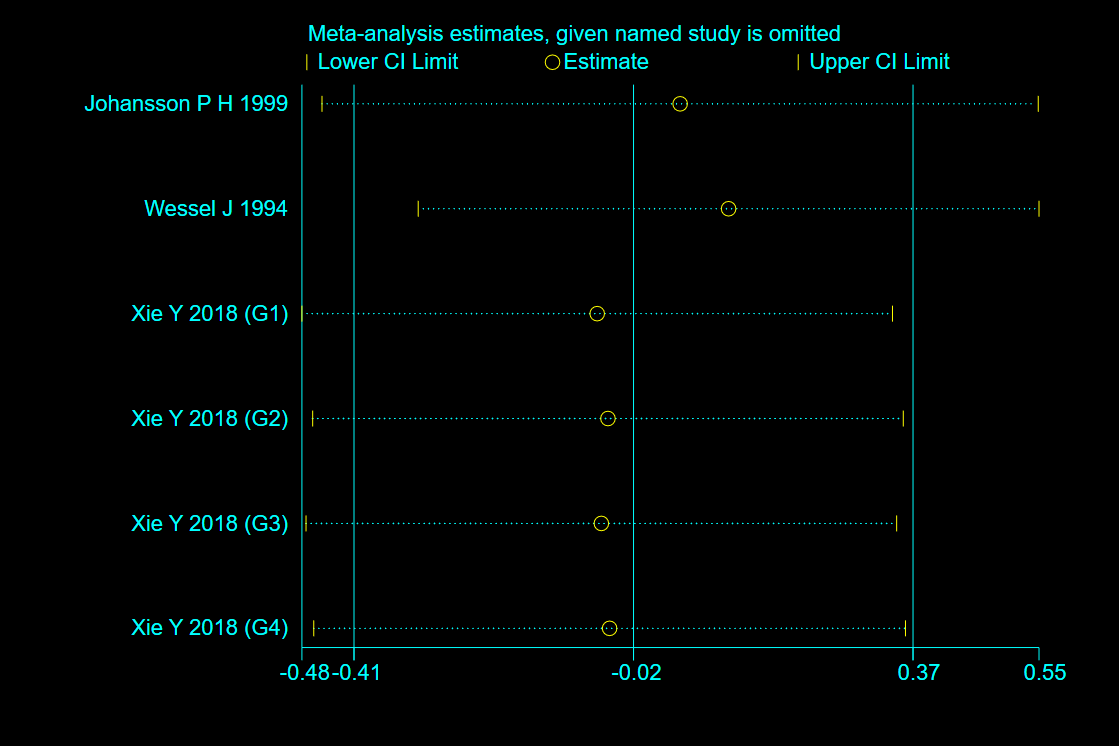


Figure 17 Leave-one-out sensitivity analysis in pain threshold.
